# Genetic investigation of sinopulmonary diseases in Vietnam: seeking specific causes from non-specific symptoms

**DOI:** 10.1186/s13023-025-04031-5

**Published:** 2025-10-15

**Authors:** Phan Thu Phuong, Nguyen Thi Le Hang, Minako Hijikata, Kozo Morimoto, Ngo Quy Chau, Le Cong Dinh, Keiko Wakabayashi, Akiko Miyabayashi, Nguyen Thu Huyen, Pham Thi Ngoc Bich, Naoto Keicho

**Affiliations:** 1 Respiratory Center, Bach Mai Hospital, Hanoi, Vietnam; 2 JIHS-BMH Medical Collaboration Center, Hanoi, Vietnam; 3 Department of Pathophysiology and Host Defense, The Research Institute of Tuberculosis, Japan Anti-Tuberculosis Association, Tokyo, Japan; 4 Respiratory Disease Center, Fukujuji Hospital, Japan Anti-Tuberculosis Association, Tokyo, Japan; 5 Tam Anh General Hospital, Hanoi, Vietnam; 6 Department of Ear, Nose, and Throat, Bach Mai Hospital, Hanoi, Vietnam; 7 ECLIPSE Project, IRD VN Social Enterprise Company Limited, Hanoi, Vietnam; 8 Family Medicine Department, Hanoi Medical University, Hanoi, Vietnam; 9 The Research Institute of Tuberculosis, Japan Anti-Tuberculosis Association, 3-1-24 Matsuyama, Kiyose, Tokyo, 204-8533 Japan; 10 Japan Institute for Health Security, Tokyo, Japan

**Keywords:** Bronchiectasis, Cystic fibrosis, Primary ciliary dyskinesia, Diffuse panbronchiolitis, Sequencing, Genetic variants

## Abstract

**Background:**

Sinopulmonary diseases are characterized by bronchiectasis (BE) and chronic rhinosinusitis, partly arising from clear genetic abnormalities such as cystic fibrosis (CF) and primary ciliary dyskinesia (PCD). However, the spectrum varies across ethnicities, and specifically, while considered rare in Southeast Asia, the current status in this region remains largely unknown. In this study, we investigated the clinical and genetic characteristics of patients with chronic symptoms affecting both the upper and lower airways in the northern region of Vietnam.

**Results:**

We recruited 200 patients with chronic rhinosinusitis and productive cough in Vietnam. Clinical characteristics including pulmonary function measurements and high-resolution chest computed tomography findings were collected. The patients’ median age was 49.0 years, with a median productive cough duration of 3 years. BE was identified in 43.8% of cases, most commonly affecting the right and left middle lung lobes (74.7% and 70.1%, respectively), and was associated with older age and bronchiolar lesions (BL). Extensive BL/BE representing 15.5% of cases (31/200), was associated with impaired pulmonary function, and seven exhibited respiratory symptoms before the age of 20. To elucidate the genetic basis of sinopulmonary diseases in patients with early onset or situs inversus, we performed genetic analyses, including targeted resequencing of genes for CF and PCD, as well as other candidate genes. Pathogenic variants identified in the *CFTR* gene were p.Trp401Ter and p.Asp979Ala only in one patient. NM_012472.6(*DNAAF11*):c.1A>G; p.Met1?, NM_080860.4(*RSPH1*):c.365+1G>A, and NM_080860.4(*RSPH1*):c.407_410del; p.Lys136MetfsTer6, all causative of PCD, were identified in the homozygous or hemizygous state in three different patients, respectively. *WFDC2* genetic abnormalities were not identified. An intron2 variant of *MUC22* (*PBMUCL1*), a candidate susceptibility gene for diffuse panbronchiolitis (DPB), was more frequently observed in patients with extensive BL/BE.

**Conclusions:**

This is the first report in Vietnamese patients with non-specific upper and lower airway symptoms to identify genetic variants specific to CF and PCD, as well as another variant potentially associated with DPB. For the future management of sinopulmonary diseases or BE with unknown causes, ethnic differences based on their genetic etiology should be carefully considered.

**Supplementary Information:**

The online version contains supplementary material available at 10.1186/s13023-025-04031-5.

## Background

Sinopulmonary diseases are assumed to share a common characteristic: a reduced mucosal defense capacity in both the upper and lower airways. In adults, this condition typically manifests as chronic rhinosinusitis (CRS) and bronchiectasis (BE), characterized by dilatation of the airways mostly with neutrophilic inflammation. Although BE can result from various etiologies [1, 2] and a standardized etiological algorithm is generally employed for the clinical determination of its causes [3], the importance of the “single-airway” concept in sinopulmonary diseases is underscored by accumulating evidence linking the common pathophysiology to genetic defects [4, 5].

Diseases in this category are often overlooked and mistaken for more common conditions like chronic obstructive pulmonary disease and asthma. They are poorly recognized, and diagnosis is generally difficult, especially in low- and middle- income countries that lack effective screening methods [6]. However, when genetically confirmed, specific diseases such as cystic fibrosis (CF) and primary ciliary dyskinesia (PCD) are expected to contribute significantly to future clinical trials, which will ultimately improve patient management.

CF [7] is an autosomal recessive disorder caused by genetic variants in the CF transmembrane conductance regulator (*CFTR*) gene, which is involved in the regulation of the water–electrolyte balance in many organs, including the upper and lower airway [8]. The most common pathogenic variant, p.Phe508del [9, 10], occurs at a frequency of 1 in 3,500–5,000 live births in European populations [8, 11]. However, CF rarely occurs in Asian populations, and a variety of rare *CFTR* genetic variants have been reported [12].

PCD is another genetic disorder that causes reduced mucociliary clearance in the respiratory epithelium, leading to recurrent infections of the upper and lower airways. The estimated incidence of PCD is 1 in 10,000 to 20,000 live births worldwide [13]. To date, more than 50 causative genes with pathogenic variants have been identified [14]. PCD is likely underdiagnosed, especially in resource-limited countries [15, 16].

Diffuse panbronchiolitis (DPB), a multifactorial disease first reported in Japan, is also classified within the spectrum of sinopulmonary diseases prevalent in eastern Asia [17]. DPB affects the respiratory bronchioles, and progresses to BE, respiratory failure, and death, if left untreated [18]. The disease is associated with human leukocyte antigen (HLA) class I alleles [19–21], and two candidate susceptibility genes located near the HLA-B locus, including *MUC22* (*PBMUCL1*), have been reported [22].

The distribution of disease-causing or disease-associated gene variants appears to be highly dependent on ethnicity [12, 23, 24]. Chronic upper and lower airway diseases commonly occur in Asian countries; however, their detailed etiology remains largely unknown. In this study, we investigated the clinical and genetic characteristics of patients with chronic symptoms affecting both the upper and lower airways in the northern region of Vietnam, and identified their causative genes, focusing on patients in whom a hereditary component was strongly suspected.

## Methods

### Study sites, patient recruitment, and sample collection

**Fig. 1:**
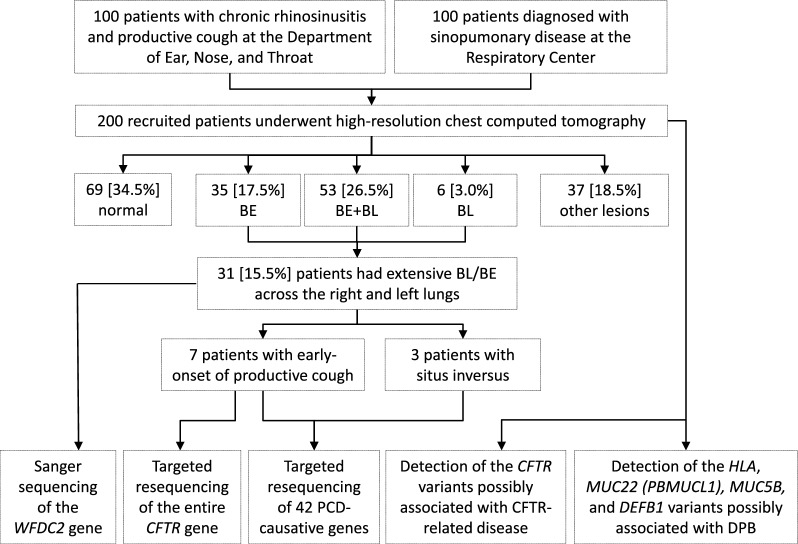
Categories of high-resolution chest CT findings and the flow of selecting patients for genetic analyses. *CT* computed tomography, *BL* bronchiolar lesions, *BE* bronchiectasis, other lesions including infiltrations, nodules, or scars, *PCD* primary ciliary dyskinesia, *DPB* diffuse panbronchiolitis

The study was conducted in Bach Mai Hospital, a central-level hospital in Hanoi, Vietnam from January 2012 to the end of February 2013. Bach Mai Hospital is the top referral hospital in Vietnam and consists of multiple centers, where patients can be referred from all northern provinces of Vietnam. Adult patients with a productive cough and who were diagnosed with CRS based on the EPOS 2007 criteria were recruited at the Department of Ear, Nose, and Throat (ENT), and those suspected of sinopulmonary diseases were recruited at the Respiratory Center. All patients underwent an interview, which included the use of a structured questionnaire, as well as physical examination, pulmonary function testing, otolaryngoscopy, high-resolution chest computed tomography (CT), and blood collection for testing for inflammatory parameters including immunoglobulin measurements, and genetic analysis (Fig. 1). Endoscopic signs of polyps, mucopurulent discharge primarily from middle meatus, or edema/mucosal obstruction primarily in middle meatus were evaluated. The chest CT findings were examined by two independent readers; discussion was undertaken and consensus made when there were any discrepancies. When a consensus was not reached, the cases were not regarded as definite findings.

### Genetic analysis to identify pathogenic variants in suspected monogenic diseases

Genomic DNA was extracted from peripheral blood cells using a QIAamp DNA Blood Mini Kit (QIAGEN). Genetic testing was performed over an extended study period and was progressively updated as new genes associated with sinopulmonary diseases were reported. The entire *CFTR* gene region was amplified using 16 overlapping long-range polymerase chain reaction (PCR) primer sets and sequenced as previously described with some modifications [25]. All products were confirmed to be of the expected sizes by agarose gel electrophoresis. The PCR primers for intron 11 were replaced with 5′-TGGGTTAGGAAGGAAGAATGCAGAGGTGG-3′ and 5′-TATGGCCAGCTTGAGACACTATGATGCCTC-3′ to amplify 13,091 bp using PrimeSTAR GXL DNA Polymerase (TaKaRa).

To determine whether the two *CFTR* variants were located in cis or trans, the corresponding region was amplified using overlapping long-range PCRs. The PCR products were sequenced using a long-read sequencer GridION (Oxford Nanopore Technologies) with ligation sequencing amplicons protocol, and reads were aligned to the human reference genome (GRCh38). Heterozygous variants were identified using FreeBayes (v1.3.2) and phased with WhatsHap (v2.8), a read-based phasing tool, using the single nucleotide variant (SNV)-only option [26], and haplotypes were determined from reads spanning multiple heterozygous sites.

Genomic DNA samples were also subjected to PCR amplification of the exon and exon–intron boundary regions of the 42 PCD causative genes (Supplementary Table S1) and sequenced using MiSeq (Illumina) as described previously [27]. Read sequences were trimmed using TrimGalore v.0.6.6 for quality control, mapped to the GRCh38 reference genome using the BWA-MEM aligner tool (v.0.7.17), and subjected to variant calls with the genomic analysis toolkit GATK (v.3.8). Finally, a VCF file including the SNV information was created using the SnpEff tool v.5.0 [28]. Pathogenicity of CF- and PCD-causing variants was interpreted based on the ACMG criteria [29] with the help of the Franklin website [30] and other databases [31, 32].

Three coding exons of *WFDC2* (exon 1–3 of NM_006103.4) were amplified by PCR, and variants were screened by Sanger sequencing (Supplementary Table S2).

### Genetic analysis of other molecules involved in airway defense mechanisms

Genetic polymorphisms known to be involved in the defense mechanisms of chronic airway infection, particularly in Asia: *HLA-A, -B, -DRB1*, *CFTR*, *MUC22*, *MUC5B* [33], and *DEFB1* [34] were chosen for association analysis of the entire cohort. DNA-based typing of *HLA-A, HLA-B*, and *HLA-DRB1* alleles was performed on the Luminex Multi-Analyte Profiling system (xMAP) with a WAKFlow HLA typing kit [35] (Wakunaga, Hiroshima, Japan). DNA fragments containing variations of the other four candidate genes were amplified by PCR and sequenced using a BigDye Terminator v.3.1 Cycle Sequencing Kit (Thermo Fisher Scientific) on a 3100 Genetic Analyzer (Thermo Fisher Scientific). The target polymorphisms and primers used for PCR amplification with direct sequencing are listed in Supplementary Table S2.

Genomic DNA samples from 506 Vietnamese healthy volunteers were used as controls [36], with the exception of HLA alleles already genotyped in 170 individuals and reported by Hoa et al. [35]. *CFTR* common variants, poly-T, TG repeats, and p.Met470Val were analyzed in 495 individuals and reported [37]. The *MUC22* intron 2 variant was genotyped in the 170 samples with known HLA genotypes.

### Statistical analysis

Chi-squared and Fisher’s exact tests were performed to compare the frequencies of events. Wilcoxon rank-sum test was used to compare nonparametric distributions between the groups. The chi-square test for the linear trend of proportion or the nonparametric test for trend, which is an extension of the Wilcoxon rank-sum test, were performed to investigate the trend across groups. Bonferroni’s correction was applied for multiple comparisons. The analyses were performed using STATA v.16 (StataCorp LLC, College Station, TX, USA). P values of < 0.05 were considered statistically significant.

## Results

### Study population

A total of 200 patients were prospectively recruited, and their median age was 49.0 years (interquartile range, IQR: 30.7–57.8). The majority of patients (194/200; 97.0%) belonged to the Vietnamese Kinh ethnicity, and female patients were predominant (135/200; 67.5%). Only 15.5% were smokers or ex-smokers, whereas 50.5% of patients had family members that smoked. Approximately half of the cohort had a self-declared allergic history (92/199; 46.2%). The median time from CRS diagnosis to recruitment was 2 (IQR: 0.25–5.00) years, and the median duration of productive cough was 3 (IQR: 1–6) years. Although patient demographics did not significantly differ between the two centers, those recruited from the Respiratory Center had experienced a longer history of productive cough than those from the ENT (3.0 years [IQR: 2.0–7.0] vs. 2.0 years [IQR: 1.0–4.8], P = 0.0086) (Table 1).

**Table 1:** Characteristics of the study population

Characteristics	All (n = 200) Freq. (%)	Department of ENT (n = 100) Freq. (%)	Respiratory center (n = 100) Freq. (%)	P value*
Demographic				
Age (median [IQR])	49.0 [30.7–57.8]	45.0 [27.6–56.2]	50.7 [33.5–56.0]	0.0525
Gender (male)	65/200 (32.5)	30/100 (30.0)	35/100 (35.0)	0.450
Body mass index (median [IQR])	21.4 [19.6–23.6]	21.9 [20.0–23.8]	21.0 [19.1–23.3]	0.1432
Kinh ethnicity	194/200 (97.0)	98/100 (98.0)	96/100 (96.0)	0.407
Occupation				
Farmer	63/200 (31.5)	30/100 (30.0)	33/100 (33.0)	0.587
Manual worker	21/200 (10.5)	13/100 (13.0)	8/100 (8.0)	
Nonmanual worker	29/200 (14.5)	16/100 (16.0)	13/100 (13.0)	
Other	87/200 (43.5)	41/100 (41.0)	46/100 (46.0)	
History				
Smoking	31/200 (15.5)	15 (15.0)	16 (16.0)	0.845
Smoking of family members	101/200 (50.5)	49/100 (49.0)	52/100 (52.0)	0.671
Allergy	92/199 (46.2)	44/99 (44.4)	48/100 (48.0)	0.615
GERD	49/194 (25.3)	22/96 (22.9)	27/98 (27.6)	0.458
Asthma	46/180 (25.6)	20/86 (23.3)	26/94 (27.7)	0.499
CRS (diagnosed)	200/200 (100.0)	100/100 (100.0)	100/100 (100.0)	
Years of suffering (median [IQR])	2.0 [0.25–5.00]	2.0 [0.5–5.5]	2.0 [0–5.0]	0.1934
Productive cough	200/200 (100.0)	100/100 (100.0)	100/100 (100.0)	
Years of suffering (median [IQR])	3.0 [1.0–6.0]	2.0 [1.0–4.8]	3.0 [2.0–7.0]	**0.0086**

^*^Comparison between the Department of ENT and the Respiratory Center

*ENT* Ear, Nose, and Throat, *IQR* interquartile range, *CRS* chronic rhinosinusitis, *GERD* gastroesophageal reflux disease

Bold shows significant P values

### Imaging patterns observed on high-resolution chest CT

The CT findings revealed no abnormalities in 34.5% (69/200) of the study cohort, whereas BE was observed in 44.0% (88/200), with the majority coexisting with bronchiolar lesions (BL) or centrilobular micronodules in 26.5% (53/200) of the cases. Among those with BE, the right and left middle lung lobes were the most commonly affected (74.7% and 70.1%, respectively), whereas the right and left upper lobes were the least (36.8% and 12.6%, respectively). BL without BE was seen in 3.0% (6/200) of the cohort, and other lesions, including infiltrations, nodules, or scars were seen in 18.5% (37/200) of the patients (Fig. 1). Patients with BE were older than those without BE (53.2 years old [IQR: 32.2–62.2] vs. 45.0 [IQR: 29.5–54.1], P = 0.0078) and had lower body mass index (BMI) and worse pulmonary function measurements. Eosinophilic endotype defined by blood eosinophil count ≥ 300 cells/µl was observed in 22.7% (20/88) of BE patients (Supplementary Table S3). Multivariable analysis showed that age and presence of BL were significantly associated with BE (adjusted odds ratios = 1.04 [95% confidence interval (CI): 1.01–1.06] and 25.11 [95% CI: 8.98–70.22], respectively), after adjustment for gender, BMI, smoking history, and duration of CRS (Table 2).

**Table 2:** Factors possibly associated with bronchiectasis based on logistic regression analysis (n = 200)

Factors	Univariate		Multivariate	
	OR	95% CI	aOR	95% CI
Age (increased by one year)	**1.02**	**1.00–1.04**	**1.04**	**1.01–1.06**
Female vs. male	0.73	0.40–1.32	0.91	0.32–2.62
Body mass index (increased by one unit)	0.84	0.76–0.93	0.91	0.79–1.04
Smoking	1.23	0.57–2.66	1.43	0.40–5.15
Years of suffering from CRS (increased by one year)	1.03	0.99–1.07	0.98	0.92–1.04
Presence of bronchiolar lesions	**26.75**	**10.59–67.59**	**25.11**	**8.98–70.22**

*aOR* adjusted odds ratio, *CRS* chronic rhinosinusitis

Bold shows significant factors

We divided the study population into three groups based on the expansion of lesions on CT: Group 1, no abnormalities; Group 2, CT findings consisting of lesions in either right or left lung, such as localized BL, BE, or other opacities, including infiltrations, nodules, or scars; and Group 3, extensive BL/BE presenting in ≥ 3 lobes across the right and left lung. The proportions of these three groups were 34.5% (69/200), 50.0% (100/200), and 15.5% (31/200), respectively (Table 3).

**Table 3:** Characteristics of patients with extensive bronchiolar lesions/bronchiectasis (BL/BE) (n = 200)

Characteristics	CT findings			P value***	P for trend****
	No abnormalities (n = 69, 34.5%) Freq. (%) or Median [IQR]	Localized* abnormalities (n = 100, 50.0%) Freq. (%) or Median [IQR]	Extensive** BL/BE (n = 31, 15.5%) Freq. (%) or Median [IQR]		
Demographic					
Age, in years	37.4 [27.5–51.0]	53.3 [36.0–61.9]	43.3 [27.6–57.8]	**0.0001**	**0.026**
Gender (male)	19/69 (27.5)	35/100 (35.0)	11/31 (35.5)	0.553	NS
Body mass index	22.4 [20.4–24.0]	21.5 [19.6–23.6]	19.9 [18.1–21.3]	**0.0004**	**0.0001**
History					
Smoking	9/69 (13.0)	20/100 (20.0)	2/31 (6.5)	0.161	NS
Allergy	39/68 (57.4)	41/100 (41.0)	12/31 (38.7)	0.075	NS
GERD	21/68 (30.9)	22/96 (22.9)	6/30 (20.0)	0.495	NS
Asthma	10/65 (15.4)	27/89 (30.3)	9/26 (34.6)	0.057	**0.0237**
CRS					
Years of suffering	2 [0–5]	1 [0–5]	3 [1–8]	0.1673	NS
Productive cough					
Years of suffering	2 [1–5]	3 [1–5]	5 [3–10]	**0.0082**	**0.0007**
Blood test					
CRP (mg/dl)	0.20 [0.10–0.50]	0.30 [0.10–1.05]	0.7 [0.4–1.6]	**0.0001**	** < 0.0001**
IgE (IU/ml)	161.7 [73.3–435.6]	240.5 [81.8–543.2]	144.4 [48.3–352.9]	0.2102	NS
Blood eosinophil count (cells/µl)					
< 50	5/69 (7.3)	10/100 (10.0)	9/31 (29.0)	**0.027**	**0.007**
50–100	13/69 (18.8)	24/100 (24.0)	5/31 (16.1)		
101–299	28/69 (40.6)	36/100 (36.0)	14/31 (45.2)		
≥ 300	23/69 (33.3)	30/100 (30.0)	3/31 (9.7)		
Rheumatic factor (U/ml)	8.2 [5.6–10.1]	10.1 [7.1–12.7]	11.9 [8.7–23.0]	**0.0001**	NS
Pulmonary function test					
VC (liter)	2.9 [2.6–3.5]	2.4 [2.1–2.9]	2.4 [1.8–2.8]	**0.0001**	** < 0.0001**
FEV1 (liter)	2.5 [2.1–2.8]	1.8 [1.4–2.3]	1.6 [1.2–2.0]	**0.0001**	** < 0.0001**
FVC (liter)	3.0 [2.6–3.6]	2.2 [2.5–3.0]	2.3 [1.8–2.7]	**0.0001**	** < 0.0001**
FEV1/FVC < 70%	7/68 (10.3%)	32/99 (32.3%)	16/31 (51.6%)	** < 0.0001**	** < 0.0001**

^*^ Localized lesions in either the right or left lung, such as BL, BE, or other lesions including infiltrations, nodules, or scars

^**^ Lesions in ≥ 3 lobes across the right and left lung

^***^ Three groups were compared

^****^ The chi-square test for linear trend of proportion or the nonparametric test for trend across ordered groups of CT findings, which is an extension of the Wilcoxon rank-sum test

*IQR* interquartile range, *CRS* chronic rhinosinusitis, *CT* computed tomography, *BL* bronchiolar lesions, *BE* bronchiectasis, *GERD* gastroesophageal reflux disease, *VC* vital capacity, *FEV1* forced expiratory volume in one second, *FVC* forced vital capacity, *NS* not significant, with 0.05 as the threshold

Bold shows significant P values

The duration of persistent productive cough was longest in patients with extensive BL/BE, followed by those with localized lesions or a normal CT (5.0 years [IQR: 3.0–10.0] vs. 3.0 years [IQR: 1.0–5.0] vs. 2.0 years [IQR: 1.0–5.0]; P = 0.0082; P value for trend = 0.0007). BMI was lowest in patients with extensive BL/BE, followed by those with localized abnormalities or normal CT (19.9 [18.1–21.3], 21.5 [19.6–23.6], and 22.4 [20.4–24.0] respectively; P = 0.0004; P value for trend = 0.0001). The levels of C-reactive protein (CRP) and rheumatoid factor were highest in patients with extensive BL/BE, followed by those with localized lesions or normal CT (0.7 mg/dL [0.4–1.6], 0.30 mg/dL [0.10–1.05], and 0.20 mg/dL [0.10–0.50], respectively; P = 0.0001 for CRP; 11.9 U/mL [8.7–23.0], 10.1 U/mL [7.1–12.7], and 8.2 U/mL [5.6–10.1], respectively; P = 0.0001 for rheumatoid factor). The proportion of blood eosinophilia (≥ 300 cells/µl) was lowest in patients with extensive BL/BE, followed by those with localized lesions and those with normal CT findings (9.7% vs. 30.0% vs. 33.3%). In contrast, the trend was reversed for eosinopenia (< 50 cells/µl) (29.0% vs. 10.0% vs. 7.3%) (P = 0.027, P value for trend = 0.007). Among the pulmonary function measurements, forced expiratory volume in one second (FEV1) was lowest in patients with extensive BL/BE, followed by those with localized CT findings or normal CT (1.6 L [1.2–2.0], 1.8 L [1.4–2.3], and 2.5 L [2.1–2.8], respectively, P = 0.0001, P value for trend < 0.0001). The proportion of a FEV1/forced vital capacity (FVC) ratio of < 0.7, indicating a pulmonary obstructive pattern, was highest among patients with extensive BL/BE, followed by those with localized abnormalities or normal CT scans (16/31, 51.6%; 32/99, 32.3%; and 7/68, 10.3%, respectively; P < 0.0001, P value for trend < 0.0001) (Table 3).

### Genetic analysis of monogenic diseases in patients with early-onset symptoms or situs inversus

Since upper and lower airway symptoms can be associated with various common respiratory diseases, careful patient selection is essential to identify inherent sinopulmonary diseases. Of the 31 patients with CT-confirmed extensive BL/BE, seven had an early onset (age < 20 years old) of productive cough, and their genomic sequences were analyzed to identify disease-causing variants in *CFTR* and PCD-related genes. In addition, three patients with situs inversus underwent a similar genetic analysis for PCD. The coding exons of *WFDC2* were analyzed in all 31 patients (Fig. 1).

#### Cystic fibrosis and other CFTR-related diseases

**Fig. 2:**
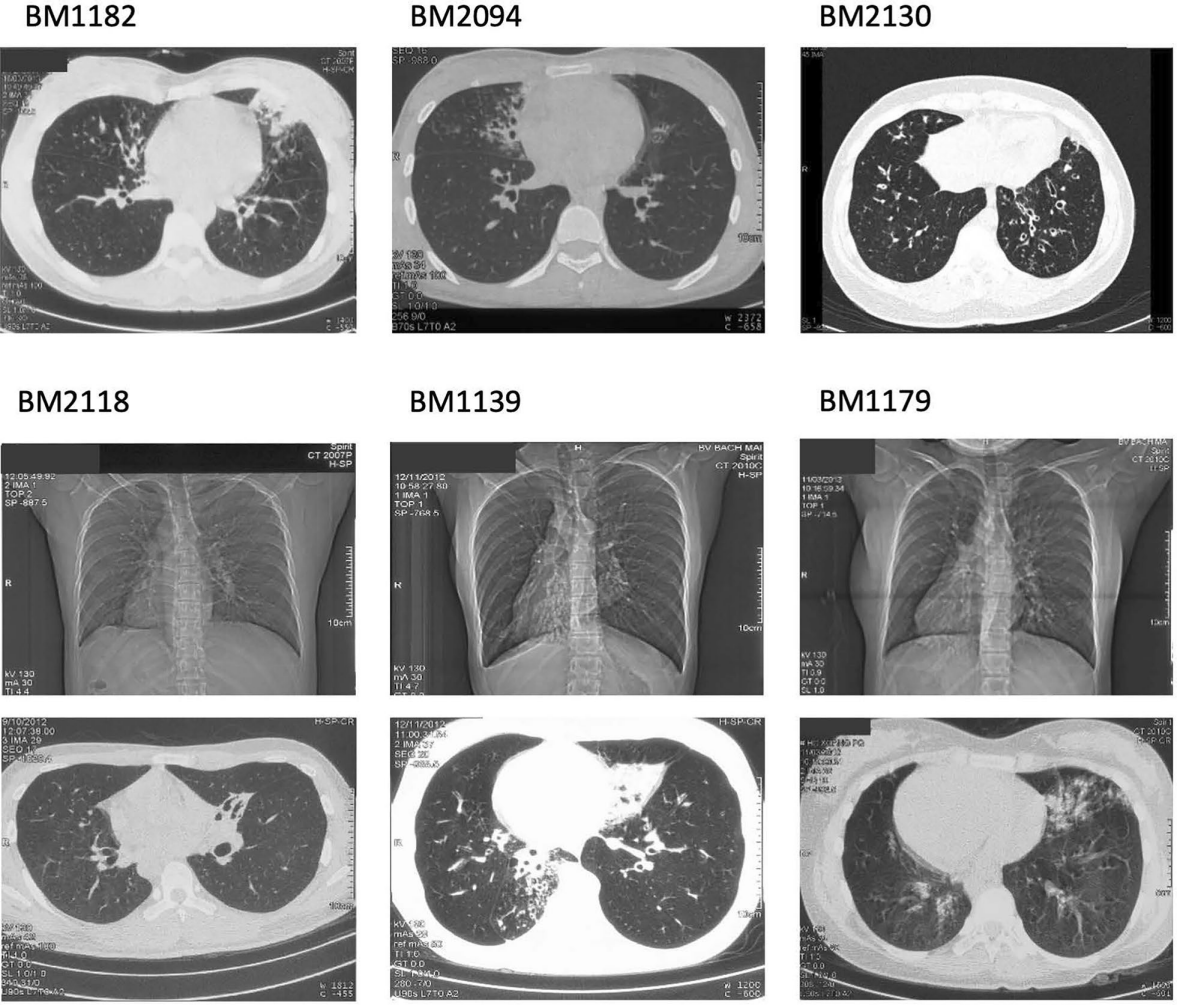
Chest CT images of patients with genetically confirmed CF or PCD, and with situs inversus. Patient BM1182 carried pathogenic *CFTR* variants. Patients BM2094, BM2130, and BM2118 had PCD-causing gene variants. Patients BM2118, BM1139, and BM1179 exhibited situs inversus. *CT* computed tomography, *CF* cystic fibrosis, *PCD* primary ciliary dyskinesia

Sequencing of the entire CFTR gene showed that one patient (BM1182) had a heterozygous pathogenic nonsense variant NM_000492.4:c.1202G>A (p.Trp401Ter) and a heterozygous nonsynonymous variant NM_000492.4:c.2936A>C (p.Asp979Ala), which is registered as pathogenic, likely pathogenic or uncertain significance in the ClinVar database and is classified as pathogenic according to American College of Medical Genetics and Genomics (ACMG) standards (Table 4). The presence of these variants was confirmed by Sanger sequencing (Supplementary Fig. S1). The 64.6 kb region from p.Trp401Ter to p.Asp979Ala was amplified using 11 overlapping long-range PCRs, and the PCR products were sequenced using a long-read sequencer. The broad and dense distribution of common heterozygous variants, which were in complete linkage disequilibrium, allowed us to phase the variants throughout the region. This confirmed that the two heterozygous variants were on different alleles (Supplementary Fig. S2 and Supplementary Table S4). This 28.5-year-old female patient had been diagnosed with CRS for more than 10 years, which had started as a persistent wet cough at age 10, with occasional hemoptysis and dyspnea (Supplementary Table S5). High-resolution chest CT revealed both BL and BE distributed in the right and left lungs (Fig. 2). Other presumably non-pathogenic missense variants and poly-T, TG repeats (TGmTn) in intron 9 identified in the seven patients tested are listed in Supplementary Table S6. Two patients harbored both Ile556Val and TG12T5.

**Table 4:** Pathogenic or likely pathogenic variants identified in *CFTR* and 42 PCD-causative genes

Gene type	Gene and variant	Variant effect	dbSNP	Location (GRCh38)	Zygosity	Pathogenicity (ClinVar* variation ID)	ACMG [30] classification	Allele frequency in gnomAD v4.1.0*	Patient, remark
CF-causing	*CFTR,* NM_000492.4:c.1202G>A (p.Trp401Ter)	Stop gained	rs397508174	chr7:117,542,101 G>A	Heterozygous	Pathogenic (53212)	Pathogenic (PM2, PVS1, PP5, PS4)	0.000	BM1182
CF-causing	*CFTR,* NM_000492.4:c.2936A>C (p.Asp979Ala)	Missense	rs397508462	chr7:117,606,701 A>C	Heterozygous	Pathogenic/Likely pathogenic/Uncertain significance (53602)	Pathogenic (PP3, PM2, PM5, PP2, PP5)	0.000008794	BM1182
PCD-causing	*DNAAF11* (*LRRC6*), NM_012472.6:c.1A>G (p.Met1?)	Start lost	NA	chr8:132,675,493 T>C	Homozygous or hemizygous**	Pathogenic (2421581)	Likely pathogenic (PS1, PM2, PVS1, PP5)	6.375e − 7	BM2118; clinically Kartagener syndrome
PCD-causing	*RSPH1*, NM_080860.4:c.365+1G>A	Splice donor	rs2054180793	chr21:42,486,370 C>T	Homozygous or hemizygous**	NA	Likely pathogenic (PVS1, PM2)	6.211e − 7	BM2094
PCD-causing	*RSPH1*, NM_080860.4:c.407_410del (p.Lys136MetfsTer6)	Frameshift	rs587777059	chr21:42,485,759 ATACT>A	Homozygous or hemizygous**	Likely pathogenic (66989)	Pathogenic (PM2, PVS1, PP5)	0.00001487	BM2130

^*^https://www.ncbi.nlm.nih.gov/clinvar/; https://franklin.genoox.com/; https://gnomad.broadinstitute.org/

^**^hemizygous: a possibility of the identified variant with an unidentified large deletion

*CF* cystic fibrosis, *PCD* primary ciliary dyskinesia, *ACMG* the American College of Medical Genetics and Genomics, *gnomAD* the Genome Aggregation Database, *NA* not available

#### PCD

For the ten patients mentioned above, sequencing of a panel of 42 PCD-related genes was conducted. In Table 4, biallelic pathogenic or likely pathogenic variants were found in two patients: patient BM2094 had a variant at splice donor site of intron 4 in *RSPH1* (NM_080860.4:c.365+1G>A); and patient BM2130 had a 4-nt deletion in exon 5 of *RSPH1*, causing a frameshift, followed by a premature termination codon (NM_080860.4:c.407_410del; p.Lys136MetfsTer6) (Table 4) (Supplementary Fig. S3). This patient also harbored Ile556Val and TG12T5, as shown in Supplementary Table S6.

Among three patients (BM1139, BM1179, and BM2118) with situs inversus, patient BM2118 had a variant in the translation start site of *DNAAF11 (LRRC6)* (NM_012472.6:c.1A>G; p.Met1?) (Table 4), but in the other two patients, BM1139 and BM1179, who are sisters from the same family, no pathogenic variants were found in our analysis. The history of repeated pulmonary infection was not clear, but all had experienced long-term productive coughs and three cases had dyspnea after exercise. The FEV1/FVC ratio was low in three patients (0.45–0.65). All chest CTs revealed BE and/or BL distributed across the right and left lungs (Fig. 2), and their BE was more frequently observed in the middle and lower lobes (Supplementary Table S7). The parent of one patient (BM2118) had CRS and all these patients were of Kinh ethnicity, the major ethnicity in Vietnam (Supplementary Table S7).

An extended genetic analysis of one patient (BM2045) with CT-based extensive BL/BE, who was not included in the initial analysis, later identified a homozygous or hemizygous frameshift variant, NM_181426.2(CCDC39):c.599_600del (p.Ile200LysfsTer8), pathogenic for PCD (Supplementary Fig. S4 and Supplementary Table S7).

#### WFDC2 genetic abnormalities

No variant was found in coding exon regions of *WFDC2* in patients with CT-based extensive BL/BE (data not shown).

### Genetic analysis of multifactorial diseases

After excluding one patient with pathogenic *CFTR* variants and three patients with PCD-causing gene variants shown above, we further investigated the distribution of known variants of other genes possibly associated with the group of 27 patients with CT-based extensive BL/BE, compared with the remaining 168 patients excluding one in which DNA was unavailable or with the healthy controls mentioned above.

#### CFTR variants possibly associated with CFTR-related disease

The frequencies of well-known *CFTR* variants, p.Phe508del, poly-T, TG repeats and c.1408A>G (p.Met470Val), were investigated in the 195 (27 + 168) patients. No p.Phe508del was detected in our Vietnamese cohort. For the remaining variants, no significant associations were shown between the CT-based extensive BL/BE group and patients in other groups or 495 controls [37] (Supplementary Table S8).

#### Genetic variants possibly associated with diffuse panbronchiolitis (DPB)

##### HLA

DNA typing of the current cohort revealed 21 HLA alleles in the A locus, 45 in the B locus, and 30 in the DRB1 locus. The frequency of the top ten HLA alleles in each locus, and those reported to be associated with DPB in Japan [20] are shown in the Supplementary Table S9. HLA-A*24:02 showed a positive association with the extensive BL/BE group compared with the remaining study population (27.8% vs. 10.7%; P = 0.001; corrected P value, Pc = 0.021); but this significance was lost when this group was compared with 170 healthy controls [35] (27.8% vs. 13.8%; P = 0.009; Pc > 0.05). HLA-B*54:01 was more frequently observed in patients with extensive BL/BE, but the significance was lost after correction of multiple comparisons. Among the HLA class II genes, HLA-DRB1*04:05 showed a positive association with the extensive BL/BE group when compared with healthy controls (14.8% vs. 3.8%; P = 0.001, Pc = 0.030), though it was not associated with the remaining study group (14.8% vs. 6.6%; P = 0.034, Pc > 0.05).

##### MUC22

The allele and genotype frequencies of an SNV in *MUC22* in intron 2, but not in exon 5, were significantly higher in patients with extensive BL/BE compared with the rest of the study population and healthy controls (0.093 vs. 0.024 and 0.018 for the T allele, P = 0.009 and 0.002, respectively; 18.5% vs. 4.8% and 3.6% for the CT genotype, P = 0.008 and 0.009, respectively) (Supplementary Table S10).

##### MUC5B and DEFB1

The allele and genotype frequencies of the insertion/deletion polymorphism of the *MUC5B* promoter (rs17235353) were not different between the extensive BL/BE group and either the rest of the study population or healthy controls (Supplementary Table S10). When similar comparisons were made for the allele frequencies of the three *DEFB1* SNVs (rs1799946, rs1800972, and rs11362), again, no differences were observed (Supplementary Table S10).

### Screening for immunoglobulin deficiency

All 31 patients with CT-based extensive BL/BE had normal IgA and IgM levels. However, one patient (BM2045) had a relatively low IgG value (7.57 g/L) (Table not shown). In this patient, the IgG4 (< 0.045 g/L) level was below the adult standard range (0.05–1.17 g/L) [38], although IgG1 (4.33 g/L), IgG2 (2.03 g/L), and IgG3 (0.72 g/L) levels were within the normal range. Unexpectedly, the patient was later identified to carry a pathogenic homozygous or hemizygous frameshift variant in *CCDC39*, as described above (Supplementary Fig. S4 and Supplementary Table S7).

## Discussion

We characterized 200 prospectively recruited patients with CRS and productive coughs at a top-level hospital in Hanoi, Vietnam. BE was observed in approximately half of the patients on CT and was associated with older age and BL. Extensive BL/BE based on CT findings was associated with clinical characteristics, including impaired pulmonary function and low BMI, along with elevation of inflammation markers, indicating that the undernutrition associated with chronic inflammation was more serious in the BE group. When focusing on ten patients with extensive BL/BE and an early onset of the disease, or situs inversus, the likely etiology was determined in four patients: one patient carried two pathogenic variants (p.Trp401Ter and p.Asp979Ala) in the *CFTR* gene, and three patients had biallelic variants in *DNAAF11* or *RSPH1*. All of these variants are relatively uncommon causes of the diseases in Western countries. In our disease association study, an intronic SNV in *MUC22*, a susceptibility to the DPB candidate gene in Asia, was also more frequently observed in the extensive BL/BE group than in the control group.

In our study, CRS frequently co-occurred with BE in older age. It is consistent with a previous systematic review reporting the prevalence of CRS among patients with BE of 62% [39]. BE is an age-associated disease, and a marked increase in prevalence is observed in the elderly [5]. Chronic BL may lead to bronchiolectasis and mucus stasis, resulting in diffuse or localized BE [40]. Blood eosinopenia (< 50 cells/μl) was more frequently observed in our patients with extensive BL/BE, which is consistent with a recent report stating that eosinopenia is associated with severity and worse outcome [41]. Among BE patients, eosinophilic endotype (≥ 300 cells/μl) was also observed in 22.7%, a group that may have an allergic component and may benefit from glucocorticoids or biologic therapies targeting type 2 inflammation [42]. The contribution of rheumatoid arthritis (RA) or other autoimmune conditions in elevating rheumatoid factors was unclear due to limited information from interviews conducted in this study. However, elevated rheumatoid factor is frequently observed in RA-associated BE [43] or patients with DPB [18].

The most frequent *CFTR* variant in European descents, p.Phe508del, was not found among our study population. This result echoes those of a review that revealed a very low frequency of this variant among Asians [12]. Instead, a heterozygous pathogenic variant (c.1202G>A, p.Trp401Ter) was found in one patient with extensive BL/BE and early onset of persistent wet cough. This is a CF-causing variant [44] with an allele frequency of 0.000106 among the *CFTR* variants identified in the CFTR2 database [7]. This patient harbored another heterozygous pathogenic variant (c.2936A>C, p.Asp979Ala) reported among Japanese CF patients [12]. The genomic positions of Trp401Ter and Asp979Ala are separated by 64,600 nt, but long-read sequencing of overlapping PCR amplicons enabled phasing of the variants [45, 46], thereby demonstrating that the two heterozygous pathogenic variants are in compound heterozygosity. Asp979Ala had a minor allele frequency of 0.005 in the Vietnamese database, which includes 406 healthy Kinh Vietnamese [47]. The p.Asp979Ala may be important as a pathogenic *CFTR* variant, particularly in the Vietnamese population.

A heterozygous c.1666A>G (p.Ile556Val) variant was also found in two patients with extensive BL/BE and early-onset cough. They also harbored the TG12T5 variant. The poly-T polymorphism (T5) observed in Asian populations, when combined with a high TG repeat, may affect the proportion of transcripts from which functional CFTR proteins can be translated [48], and presumably leads to impaired mucociliary clearance in the airway. Although the association between Ile556Val and CF remains undefined, Ile556Val has been reported as the most frequent *CFTR* variation in Chinese children with CF [49]. Although this variant is common and benign when occurring alone, the co-occurrence of this mutation with TG12T5 and a PCD-causing variant in patients with extensive BL/BE may be uncommon and deserves further investigation.

Reports on PCD from non-Western countries have increased in recent years, including from resource-limited settings [50–52]. *RSPH1* variants affect the radial spoke component of cilia, causing PCD [53, 54]. The *RSPH1* variants c.365+1G>A and c.407_410del observed in our study are rare in the Genome Aggregation Database [32] and have not been registered in the database of the Vietnamese Kinh population [47], although c.407_410del has been reported once in a PCD patient of European origin [53].

*DNAAF11* or *LRRC6* encodes a leucine-rich-repeat (LRR)-containing protein that is involved in the cytoplasmic dynein axonemal assembly of cilia [55]. Although *DNAAF11* variants are relatively rare as a cause of PCD in Western countries [56, 57], they are a common cause of PCD with laterality defects in South Asia [58]. The *DNAAF11* c.1A>G (p.Met1?) variant detected in our study is rare; it has only one submission in the ClinVar database and is not found in the population genome data, including the Vietnamese database [47]. This variant (ATG>GTG) disrupts translation initiation as GTG is not usually used as a start codon in eukaryotes. This is supported by the pathogenic variant NM_000492.4:c.1A>G (p.Met1Val). The patient had mild BL confirmed via CT with slightly elevated inflammation markers, and his pulmonary function was not severely affected. In addition, he was fertile with three children, suggesting a mild disease phenotype.

Remarkably, PCD caused by *DNAH5, DNAI1*, and *CCDC40* variants, which are common in European populations [59], were not identified even in patients with situs inversus in our study. Recently in Vietnam, three patients with Kartagener syndrome were reported [60], but no genetic investigations have been reported so far.

A novel disease caused by *WFDC2* variants has recently been identified in patients with severe respiratory disorders, such as BE in all lung fields, chronic rhinosinusitis, and polyps resembling CF [61]. No abnormal *WFDC2* variants were found in 31 patients with extensive BL/BE.

DPB is a chronic airway disease associated with HLA in Asians, for example, HLA-B54 among the Japanese [20]. In our study, HLA-B*54:01 tended to be more frequently seen in patients with extensive BL/BE excluding CF and PCD, but it did not reach significant levels when considering multiple comparisons. A*24:02 also tended to be associated with BL/BE in our study population. HLA-A24 involvement has been reported in Chinese DPB patients [62, 63], but the significance of this association remains unclear. The increase observed in the frequency of HLA-DRB1*04:05 was presumably due to linkage disequilibrium with other class I alleles.

The genotype and allele frequencies of *MUC22* intron 2 SNVs located near the *HLA-B* locus were significantly higher in patients with extensive BL/BE compared with controls. The associations of the same SNVs with the disease were observed both in Japanese DPB patients [22] and in this Vietnamese study. However, because of the strong linkage disequilibrium in the HLA region, the causative gene locus has not been determined. Nevertheless, it would be interesting if a common genetic predisposition in Asians underlies susceptibility to chronic airway inflammatory diseases in the Vietnamese and Japanese.

MUC5B is one of the major secreted mucins in the airways. A polymorphism in the promoter region of *MUC5B* is associated with DPB in the Japanese population [33], and a splicing event in this gene leading to MUC5B absence has been reported in a Canadian family case series with respiratory diseases [64]. Human beta-defensins are members of a large family of antimicrobial peptides. An SNV in the encoding gene, *DEFB1*, is associated with chronic lung disease [34].

This study found that a relatively low IgG level was observed in one patient with extensive BL/BE, although genetic testing incidentally identified a biallelic PCD-causing variant, NM_181426.2(*CCDC39*):c.599_600del. Measuring IgG subclasses should be considered when other causes of BE have been ruled out, since immunoglobulin replacement therapy may improve short- and long-term outcomes [65].

Our study had some limitations. Nongenetic screening tests, such as the sweat chloride test for CF and electron microscopy or high-speed video microscopy for PCD, were not available in our local setting during the study period. However, these diseases have recently been diagnosed with genetic tests, the gold standard for diagnosing these diseases. Genetic testing was conducted over time and updated as additional sinopulmonary disease genes were identified. Despite the moderate sample size recruited in a single central hospital, for the first time in Vietnam, various distinctive pathogenic variants responsible for sinopulmonary diseases were identified. This study focused on the genetic investigation of bronchiectasis, including CF, PCD, and DPB. However, genes associated with immunodeficiency, as well as other etiologies, including connective tissue diseases remain underexplored due to limited on-site resources. In particular, when an underlying primary immunodeficiency is suspected, the large number of potential causative genes poses a significant challenge to achieving a definitive genetic diagnosis.

Family segregation analysis, a common practice in pediatric studies, was not planned in the original study, and asking for details about privacy concerns, including the parents' marital status, was not included in the questionnaire approved by the local Ethical Committees. This is a common challenge when dealing with adult patients, especially in medical settings where genetic testing has not yet been routinely incorporated into clinical practice.

In our study, most of the patients (97.0%) were from Kinh ethnic families, the major ethnicity in Vietnam, where consanguineous marriage is believed to be rare. Nevertheless, the frequent occurrence of apparently homozygous PCD cases in this study highlights the need to clarify the real frequency of consanguineous marriage and the possibility of founder variants in the Vietnamese population. Future nationwide investigations to precisely assess other, nongenetic causes of BE and BL, such as post-infection, autoimmune, or allergic diseases are warranted.

## Conclusions

This study identified CF- and PCD-causing genetic variants, as well as a possible association with DPB susceptibility genes, in a cohort of Vietnamese patients with CRS and productive cough, deepening our understanding of ethnic differences through the genetic spectrum of sinopulmonary diseases. Our study highlighted the importance of investigating specific causes underlying non-specific symptoms when considering future treatment strategies. Considering the rapid development of modern genetic sequencing technology, now is the time to prepare and develop a system for the definite diagnosis and management of these airway diseases.

## Supplementary Information

Supplementary material 1. Table S1: PCD-causative genes analyzed in the current study. PCD: primary ciliary dyskinesia; CILD: ciliary dyskinesia; OMIM: Online Mendelian Inheritance in Man (https://www.omim.org/). Table S2: Primers used for PCR amplification and direct sequencing. Table S3: Characteristics of patients with bronchiectasis (n = 200). *Chi-square test, Fisher’s exact test, or Wilcoxon rank-sum test. IQR: interquartile range, CRS: chronic rhinosinusitis, CT: computed tomography, GERD: gastroesophageal reflux disease, VC: vital capacity, FEV1: forced expiratory volume in one second, FVC: forced vital capacity, bold shows significant P values. Table S4: Experimentally resolved *CFTR* haplotypes in BM1182. Table S5: Characteristics of Patient BM1182 with pathogenic *CFTR* variants detected by sequencing of the entire gene. CRS: chronic rhinosinusitis, GERD: gastroesophageal reflux disease, CT: computed tomography, VC: vital capacity, FEV1: forced expiratory volume in one second, FVC: forced vital capacity. Table S6: Benign, likely benign, or variants of uncertain significance identified by sequencing of the entire* CFTR* gene among cases with extensive BL/BE and early-onset, persistent productive cough (n = 7). M: male, F: female, BL: bronchiolar lesions, BE: bronchiectasis. *https://www.ncbi.nlm.nih.gov/clinvar/. Table S7: Characteristics of patients with suspected PCD with or without identified causative genes using targeted resequencing of 42 PCD-causative genes. *Sisters with each other. PCD: primary ciliary dyskinesia, CRS: chronic rhinosinusitis, VC: vital capacity, FEV1: forced expiratory volume in one second, FVC: forced vital capacity, CT: computed tomography, GERD: gastroesophageal reflux disease, NA: not available. **Although this patient was not initially a candidate for PCD screening, genetic testing prompted by low IgG levels incidentally identified a PCD-causing variant. Table S8: *CFTR* variants possibly associated with extensive BL/BE. *Current study population, after excluding one cystic fibrosis and three primary ciliary dyskinesia genetically confirmed cases and one in which DNA was unavailable. **495 Vietnamese healthy controls (Nam MH, Am J Med Genet A. 2005). BL: bronchiolar lesions, BE: bronchiectasis, CT: computed tomography. Table S9: Frequencies of HLA alleles† in extensive BL/BE compared with controls. † Ten HLA alleles with the highest frequencies in each locus, and those reported to be associated with DPB in Japan (Keicho, Am J Respir Crit Care Med. 1998) are shown. ¶ Current study population, after excluding one case in which the DNA sample was unavailable, one cystic fibrosis and three primary ciliary dyskinesia genetically confirmed cases. § 170 Vietnamese healthy control (Hoa BK, Tissue Antigen. 2008). BL: bronchiolar lesions, BE: bronchiectasis, CT: computed tomography, bold shows significant P value after Bonferroni correction, NS: not significant. Table S10: Genes and single nucleotide variants possibly associated with extensive BL/BE*. *After excluding one cystic fibrosis and three primary ciliary dyskinesia genetically confirmed cases. **Current study population, after excluding one cystic fibrosis and three primary ciliary dyskinesia genetically confirmed cases, and one in which DNA was unavailable. ***168 (Hoa BK, Tissue Antigen. 2008) and 506 Vietnamese healthy volunteers (Hijikata M. Hum Genet. 2012) were used as controls. BL: bronchiolar lesions, BE: bronchiectasis, CT: computed tomography, I: insertion, D: deletion, bold shows significant P values. Fig. S1: The pathogenic *CFTR* variants in Patient BM1182. The whole-*CFTR*-gene sequencing was performed using a next-generation sequencer, and the reads were mapped to the human reference genome GRCh38 to identify the variants (left panel). The variants were confirmed by PCR-direct Sanger sequencing (right panel). The results from a control sample are also shown. Arrows indicate the positions of the variants. (a) A heterozygous pathogenic variant NM_000492.4:c.1202G>A (p.Trp401Ter); chr7:117,542,101 G>A; rs397508174. (b) A heterozygous pathogenic variant NM_000492.4:c.2936A>C (p.Asp979Ala); chr7:117,606,701 A>C; rs397508462. PCR: polymerase chain reaction. Fig. S2: Overlapping long-range PCRs in the *CFTR* region and phased long reads in Patient BM1182. The genomic region spanning *CFTR* :c.1202G>A (p.Trp401Ter) and c.2936A>C (p.Asp979Ala) was amplified using 11 overlapping long-range PCRs (upper panel), sequenced with a long-read sequencer, and reads were aligned to the human reference genome. Haplotype tags were assigned to each read, and single nucleotide variants with the two haplotypes (haplotype 1 in light blue and haplotype 2 in pink) were visualized in IGV (lower panel). PCR: polymerase chain reaction, IGV: Integrative Genomics Viewer. Fig. S3: PCD-causing gene variants identified by next-generation sequencing (left panel) and PCR-direct Sanger sequencing (right panel). (a) A homozygous/hemizygous pathogenic variant to disrupt the start codon of *DNAAF11* from ATG to GTG was identified in Patient BM2118. NM_012472.6:c.1A>G (NP_036604.2:p.Met1?); chr8:132,675,493 T>C. (b) A homozygous/hemizygous pathogenic variant to disrupt the splice donor sequence of intron 4 from GT to AT in the *RSPH1* gene of Patient BM2094: NM_080860.4:c.365+1G>A; chr21:42,486,370 C>T. (c) A homozygous/hemizygous pathogenic variant, a four-nucleotide deletion causing a frameshift and premature stop codon in *RSPH1* of Patient BM2130: NM_080860.4:c.407_410del (NP_543136.1:p.Lys136MetfsTer6). PCD: primary ciliary dyskinesia, PCR: polymerase chain reaction. Fig. S4: PCD-causing *CCDC39* variant in Patient BM2045. PCD-causing gene variants identified by next-generation sequencing (left panel) and PCR-direct Sanger sequencing (right panel) in Patient BM2045, compared with a control sample. Arrows indicate the positions of the homozygous/hemizygous pathogenic variant, a two-nucleotide deletion that causes a frameshift and premature stop codon in *CCDC39* : NM_181426.2:c.599_600del (NP_852091.1:p.Ile200LysfsTer8). PCD: primary ciliary dyskinesia, PCR: polymerase chain reaction.

## Data Availability

All data pertaining to the manuscript have been provided in the forms of tables and figures. Supporting information is available as Supplementary Tables S1–S10 and Supplementary Figures S1–S4. Datasets pertaining to the sequence searches described here are available from the corresponding author on request.
