# Supplementary material for: Genetic investigation of sinopulmonary diseases in Vietnam: seeking specific causes from non-specific symptoms
Source: Orphanet J Rare Dis. 2025 Oct 15;20:517. doi: 10.1186/s13023-025-04031-5 (PMC12522257; doi:10.1186/s13023-025-04031-5)
Supplement: Supplementary file 1 — Supplementary material 1. Table S1: PCD-causative genes analyzed in the current study. PCD: primary ciliary dyskinesia; CILD: ciliary dyskinesia; OMIM: Online Mendelian Inheritance in Man (https://www.omim.org/). Table S2: Primers used for PCR amplification and direct sequencing. Table S3: Characteristics of patients with bronchiectasis (n = 200). *Chi-square test, Fisher’s exact test, or Wilcoxon rank-sum test. IQR: interquartile range, CRS: chronic rhinosinusitis, CT: computed tomography, GERD: gastroesophageal reflux disease, VC: vital capacity, FEV1: forced expiratory volume in one second, FVC: forced vital capacity, bold shows significant P values. Table S4: Experimentally resolved CFTR haplotypes in BM1182. Table S5: Characteristics of Patient BM1182 with pathogenic CFTR variants detected by sequencing of the entire gene. CRS: chronic rhinosinusitis, GERD: gastroesophageal reflux disease, CT: computed tomography, VC: vital capacity, FEV1: forced expiratory volume in one second, FVC: forced vital capacity. Table S6: Benign, likely benign, or variants of uncertain significance identified by sequencing of the entire CFTR gene among cases with extensive BL/BE and early-onset, persistent productive cough (n = 7). M: male, F: female, BL: bronchiolar lesions, BE: bronchiectasis. *https://www.ncbi.nlm.nih.gov/clinvar/. Table S7: Characteristics of patients with suspected PCD with or without identified causative genes using targeted resequencing of 42 PCD-causative genes. *Sisters with each other. PCD: primary ciliary dyskinesia, CRS: chronic rhinosinusitis, VC: vital capacity, FEV1: forced expiratory volume in one second, FVC: forced vital capacity, CT: computed tomography, GERD: gastroesophageal reflux disease, NA: not available. **Although this patient was not initially a candidate for PCD screening, genetic testing prompted by low IgG levels incidentally identified a PCD-causing variant. Table S8: CFTR variants possibly associated with extensive BL/BE. *Current stu [file 13023_2025_4031_MOESM1_ESM.pdf]

Genetic investigation of sinopulmonary diseases in Vietnam: seeking specific causes from non-specific symptoms

Phan Thu Phuong[1,§], Nguyen Thi Le Hang[2,§], Minako Hijikata[3], Kozo Morimoto[4], Ngo Quy Chau[5], Le Cong Dinh[6], Keiko Wakabayashi[3], Akiko Miyabayashi[3], Nguyen Thu Huyen[2,7], Pham Thi Ngoc Bich[2,8], Naoto Keicho[9,10*]

[1] Respiratory Center, Bach Mai Hospital, Hanoi, Vietnam.
[2] JIHS-BMH Medical Collaboration Center, Hanoi, Vietnam.
[3] Department of Pathophysiology and Host Defense, The Research Institute of Tuberculosis, Japan Anti-Tuberculosis Association, Tokyo, Japan.
[4] Respiratory Disease Center, Fukujuji Hospital, Japan Anti-Tuberculosis Association, Tokyo, Japan.
[5] Tam Anh General Hospital, Hanoi, Vietnam.
[6] Department of Ear, Nose, and Throat, Bach Mai Hospital, Hanoi, Vietnam.
[7] ECLIPSE project, IRD VN Social Enterprise Company Limited, Hanoi, Vietnam.
[8] Family Medicine Department, Hanoi Medical University, Hanoi, Vietnam.
[9] The Research Institute of Tuberculosis, Japan Anti-Tuberculosis Association, Tokyo, Japan.
[10] Japan Institute of Health Security, Tokyo, Japan.
[§] Phan Thu Phuong and Nguyen Thi Le Hang contributed equally to this work and share first authorship.

***Corresponding author**
Naoto Keicho, MD, PhD
Vice Director
The Research Institute of Tuberculosis
Japan Anti-Tuberculosis Association
3-1-24 Matsuyama, Kiyose, Tokyo 204-8533, JAPAN
E-mail: nkeicho-tky@umin.ac.jp

Supporting information:
Supplementary Tables S1–S10
and
Supplementary Figures S1–S4.

Supplementary Table S1: PCD-causative genes analyzed in the current study

No	OMIM registration	Gene name	Alias	Location
1	CILD1 (244400)	*DNAI1*		9p13
2	CILD2 (606763)	*DNAAF3*	*C19orf51*	19q13
3	CILD3 (608644)	*DNAH5*		5p15
4	CILD5 (608647)	*HYDIN*		16q22.2
5	CILD6 (610852)	*NME8*	*TXNDC3*	7p14
6	CILD7 (611884)	*DNAH11*		7p15
7	CILD9 (612444)	*DNAI2*		17q25
8	CILD10 (612518)	*DNAAF2*	*KTU*	14q21
9	CILD11 (612649)	*RSPH4A*		6q22
10	CILD12 (612650)	*RSPH9*		6p21
11	CILD13 (613193)	*DNAAF1*	*LRRC50*	16q24
12	CILD14 (613807)	*CCDC39*		3q26
13	CILD15 (613808)	*CCDC40*		17q25
14	CILD16 (614017)	*DNAL1*		14q24
15	CILD17 (614679)	*CCDC103*		17q21
16	CILD18 (614874)	*DNAAF5*	*HEATR2*	7p22
17	CILD19 (614935)	*DNAAF11*	*LRRC6*	8q24
18	CILD20 (615067)	*ODAD1*	*CCDC114*	19q13
19	CILD21 (615294)	*DRC1*	*CCDC164*	2p23
20	CILD22 (615444)	*ZMYND10*	*DNAAF7*	3p21
21	CILD23 (615451)	*ODAD2*	*ARMC4*	10p12
22	CILD24 (615481)	*RSPH1*		21q22
23	CILD25 (615482)	*DNAAF4*	*DYX1C1*	15q21
24	CILD26 (615500)	*CFAP298*	*C21orf59*	21q22
25	CILD27 (615504)	*CCDC65*	*DRC2*	12q13
26	CILD28 (615505)	*SPAG1*	*DNAAF13*	8q22
27	CILD29 (615872)	*CCNO*		5q11
28	CILD30 (616037)	*ODAD3*	*CCDC151*	19p13
29	CILD32 (616481)	*RSPH3*		6q25
30	CILD33 (616726)	*GAS8*	*DRC4*	16q24
31	CILD34 (617091)	*DNAJB13*		11q13
32	CILD35 (617092)	*ODAD4*	*TTC25*	17q21
33	CILD36 (300991)	*DNAAF6*	*PIH1D3*	Xq22
34	CILD38 (618063)	*CFAP300*	*C11orf70*	11q22
35	CILD39 (618254)	*LRRC56*		11p15
36	CILD41 (618449)	*GAS2L2*		17q12
37	CILD42 (618695)	*MCIDAS*		5q11
38	CILD43 (618699)	*FOXJ1*		17q25
39	CILD45 (618801)	*TTC12*		11q23
40	CILD46 (619436)	*STK36*		2q35
41	311200	*OFD1*		Xp22
42	618704	*CFAP221*	*PCDP1*	2q14

PCD: Primary ciliary dyskinesia; CILD: ciliary dyskinesia
OMIM: Online Mendelian Inheritance in Man (https://www.omim.org)

Supplementary Table S2: Primers used for PCR amplification and direct sequencing

Gene	Target polymorphsims	Primers	Primer sequence 5´-3´	Amplified genomic region and PCR product size	PCR Enzyme for amplification
CFTR	Poly-T tract in intron 9 near the splice-acceptor site of exon 10 (NM_000492.4:c.1210-12_1210-6, rs1805177)	PCR forward primer<br>PCR reverse primer<br>Sequencing primer	gatcatgtcctctagaaaccg<br>ggagaagaggatgaccactg<br>cagcaaccgccaacaactgtcctc	chr7:117548450-117549361, 912bp	KOD FX Neo (Toyobo Life Science)
DEFB1	Three SNPs in the promoter region (rs1799946, rs1800972 and rs11362, located at positions -52, -44, and -20 of the transcription start site, respectively)	PCR forward primer<br>PCR reverse primer<br>Sequencing primer	acccttgactgtggcacctc<br>gggatgggaaactctagcag<br>acccttgactgtggcacctc	chr8:6877763-6878025, 263 bp	AmpliTaq Gold DNA polymerase (Thermo Fisher Scientific)
MUC5B	Insertion/deletion polymorphism in the promoter region (rs17235353)	PCR forward primer<br>PCR reverse primer<br>Sequencing primer 1<br>Sequencing primer 2	aacagacctggacaccaccctag<br>atccaggacactcagggagtcgta<br>aacagacctggacaccaccctag<br>ttacctgcctgcggcaccacgagcat	chr11:1221884-1223324, 1441 bp	KOD FX Neo (Toyobo Life Science)
MUC22	SNP in intron 2 (NM_001395414.1:c.71-1962G>A, rs117121291)	PCR forward primer<br>PCR reverse primer<br>Sequencing primer	tttcttttgctgacaggggtatactcagag<br>tacaggcgtgaatcgacaggcaagactgac<br>ttcatcatgattaggctcatg	chr6:31022430-31024591, 2162 bp	KOD FX Neo (Toyobo Life Science)
	SNP in exon 5 (NM_001395414.1:c.5134A>G, NP_001382343.1:p.Asn1712Asp, rs4248153)	PCR forward primer<br>PCR reverse primer<br>Sequencing primer	gcttcctatgctccatttta<br>gtatggtgattagggagagg<br>gtatggtgattagggagagg	chr6:31034608-31035101, 494 bp	KOD FX Neo (Toyobo Life Science)
WFDC2	Exons 1 and 2	PCR forward primer<br>PCR reverse primer<br>Sequencing primer 1<br>Sequencing primer 2	gcggttaaatccccgcacctgagc<br>agcccgtcatgcaccagagactcg<br>ctctccacctccagcacattggac<br>actgacggatctggtttcaaccgc	chr20:45469721-45470783, 1063 bp	KOD FX Neo (Toyobo Life Science)
	Exon 3	PCR forward primer<br>PCR reverse primer<br>Sequencing primer 1	gatcttcctgggcctcctgagagc<br>ctaacctcccttcacctccgcctg<br>ccgcctgggtgacgttttctcctg	chr20:45479772-45480186, 415 bp	KOD FX Neo (Toyobo Life Science)

Supplementary Table S3: Characteristics of patients with bronchiectasis (n = 200)

**Characteristics**	**CT findings**		
	**Without bronchiectasis (n = 112, 56.0%)** Freq. (%) or Median [IQR]	**With bronchiectasis (n = 88, 44.0%)** Freq. (%) or Median [IQR]	**P value***
Demographic			
Age, in years	45.0 [29.5–54.1]	53.2 [32.2–62.2]	**0.0078**
Gender (male)	79/112 (70.5)	56/88 (63.6)	0.3011
Body mass index	22.4 [20.3–23.9]	20.7 [18.8–23.1]	**0.0005**
History			
Smoking	16/112 (14.3)	15/88 (17.1)	0.5924
Allergy	60/111 (54.1)	32/88 (36.4)	**0.0129**
GERD	30/108 (27.8)	19/86 (22.1)	0.3653
Asthma	19/101 (18.8)	27/79 (34.2)	**0.0190**
Pneumonia	7/106 (6.6)	26/80 (32.5)	**< 0.0001**
CRS (diagnosed)			
Years of suffering (median [IQR])	2 [0.5–5]	2 [0–7]	0.6768
Productive cough			
Years of suffering (median [IQR])	2 [1–4]	4 [2–10]	**0.0001**
Dyspnoea	41/112 (36.6)	59/88 (67.1)	**< 0.0001**
Hemotysis	10/112 (8.9)	26/88 (30.0)	**0.0002**
Positive sputum culture	15/107 (14.0)	15/84 (17.9)	0.4693
Blood test			
CRP (mg/dl)	0.20 [0.10–0.50]	0.50 [0.15–1.60]	**0.0002**
IgE (IU/ml)	191.3 [75.3–486.6]	199.4 [63.1–429.3]	0.6176
Blood eosinophil count (cells/μl)			
< 50	9/112 (8.0)	15/88 (17.1)	0.157
50-100	22/112 (19.6)	20/88 (22.7)	
101-299	45/112 (40.2)	33/88 (37.5)	
≥ 300	36/112 (32.1)	20/88 (22.7)	
Rheumatic factor (U/ml)	9.3 [6.0–11.5]	11.2 [7.0–16.4]	**0.0098**
Pulmonary function test			
VC (liter)	2.8 [2.4–3.3]	2.4 [1.8–2.7]	**< 0.0001**
FEV1 (liter)	2.3 [1.9–2.8]	1.7 [1.2–2.1]	**< 0.0001**
FVC (liter)	2.9 [2.5–3.4]	2.3 [1.8–2.8]	**< 0.0001**
FEV1/FVC < 70%	19/111 (17.1)	36/87 (41.4)	**0.0002**

*Chi-square test, Fisher's exact test, or Wilcoxon rank-sum test.

IQR: inter quartile range, CRS: chronic rhinosinusitis, CT: computed tomography, GERD: gastroesophageal reflux disease, VC: vital capacity, FEV1: forced expiratory volume in one second, FVC: forced vital capacity, **bold** shows significant P value.

Supplementary Table 4: Experimentally resolved *CFTR* haplotypes in BM1182

<table>
<tr><th>Chromosome</th><th>Position</th><th>Reference allele</th><th>Alternative allele</th><th>Phasing</th><th>Haplotype-1</th><th>Haplotype-2</th><th>dbSNP ID</th><th>HGVS.c</th><th>HGVS.p</th><th>gnomAD EAS MAF</th><th>PCR range (Iso M, et al.)</th><th>PCR range (junction)</th><th>Primer name (junction)</th><th>Primer sequence (5'→3')</th></tr>
<tr><td>7</td><td>117538154</td><td>A</td><td>G</td><td>0|1</td><td>A</td><td>G</td><td>rs213935</td><td>c.869+1481A>G</td><td></td><td>0.418861</td><td>PCR-6</td><td></td><td></td><td></td></tr>
<tr><td>7</td><td>117541098</td><td>A</td><td>G</td><td>0|1</td><td>A</td><td>G</td><td>rs2237725</td><td>c.1116+752A>G</td><td></td><td>0.413953</td><td>|</td><td></td><td></td><td></td></tr>
<tr><td>7</td><td>117541455</td><td>G</td><td>A</td><td>0|1</td><td>G</td><td>A</td><td>rs4148703</td><td>c.1117-561G>A</td><td></td><td>0.412277</td><td>|</td><td></td><td></td><td></td></tr>
<tr><td>7</td><td>117542101</td><td>G</td><td>A</td><td>1|0</td><td>A</td><td>G</td><td>rs397508174</td><td>c.1202G>A</td><td>p.Trp401Ter</td><td>NA</td><td>|</td><td></td><td></td><td></td></tr>
<tr><td>7</td><td>117544462</td><td>G</td><td>A</td><td>0|1</td><td>G</td><td>A</td><td>rs213936</td><td>c.1209+2354G>A</td><td></td><td>0.419243</td><td>|</td><td></td><td></td><td></td></tr>
<tr><td>7</td><td>117545017</td><td>T</td><td>C</td><td>0|1</td><td>T</td><td>C</td><td>rs213937</td><td>c.1209+2909T>C</td><td></td><td>0.417896</td><td>|</td><td></td><td></td><td></td></tr>
<tr><td>7</td><td>117546641</td><td>G</td><td>A</td><td>0|1</td><td>G</td><td>A</td><td>rs34456592</td><td>c.1210-2000G>A</td><td></td><td>0.412800</td><td>|</td><td></td><td></td><td></td></tr>
<tr><td>7</td><td>117547407</td><td>T</td><td>C</td><td>0|1</td><td>T</td><td>C</td><td>rs12533524</td><td>c.1210-1234T>C</td><td></td><td>0.418470</td><td>|</td><td>J6-7</td><td>F6-7_J_s1</td><td>ACAACAGTCTTCCACTGGTACAAGTGC</td></tr>
<tr><td>7</td><td>117547605</td><td>T</td><td>C</td><td>0|1</td><td>T</td><td>C</td><td>rs2027944</td><td>c.1210-1036T>C</td><td></td><td>0.418017</td><td>|</td><td>|</td><td></td><td></td></tr>
<tr><td>7</td><td>117547768</td><td>G</td><td>A</td><td>0|1</td><td>G</td><td>A</td><td>rs2027945</td><td>c.1210-873G>A</td><td></td><td>0.416376</td><td>|</td><td>|</td><td></td><td></td></tr>
<tr><td>7</td><td>117549218</td><td>G</td><td>A</td><td>0|1</td><td>G</td><td>A</td><td>rs1820871</td><td>c.1392+395G>A</td><td></td><td>0.418185</td><td>PCR-6</td><td>|</td><td></td><td></td></tr>
<tr><td>7</td><td>117550544</td><td>T</td><td>C</td><td>0|1</td><td>T</td><td>C</td><td>rs1896888</td><td>c.1392+1721T>C</td><td></td><td>0.416344</td><td>PCR-7</td><td>|</td><td></td><td></td></tr>
<tr><td>7</td><td>117551229</td><td>G</td><td>A</td><td>0|1</td><td>G</td><td>A</td><td>rs213946</td><td>c.1392+2406G>A</td><td></td><td>0.418403</td><td>|</td><td>|</td><td></td><td></td></tr>
<tr><td>7</td><td>117551765</td><td>T</td><td>C</td><td>0|1</td><td>T</td><td>C</td><td>rs10953847</td><td>c.1392+2942T>C</td><td></td><td>0.418344</td><td>|</td><td>J6-7</td><td>F6-7_J_as1</td><td>GCAACAGCACTGCTTCAGCTGTGATGC</td></tr>
<tr><td>7</td><td>117552706</td><td>A</td><td>C</td><td>0|1</td><td>A</td><td>C</td><td>rs739378</td><td>c.1392+3883A>C</td><td></td><td>0.416050</td><td>|</td><td></td><td></td><td></td></tr>
<tr><td>7</td><td>117553882</td><td>A</td><td>G</td><td>0|1</td><td>A</td><td>G</td><td>rs7803222</td><td>c.1392+5059A>G</td><td></td><td>0.412141</td><td>|</td><td></td><td></td><td></td></tr>
<tr><td>7</td><td>117556314</td><td>C</td><td>T</td><td>0|1</td><td>C</td><td>T</td><td>rs2027946</td><td>c.1393-3150C>T</td><td></td><td>0.412294</td><td>|</td><td></td><td></td><td></td></tr>
<tr><td>7</td><td>117559403</td><td>A</td><td>G</td><td>0|1</td><td>A</td><td>G</td><td>rs34855237</td><td>c.1393-61A>G</td><td></td><td>0.417442</td><td>|</td><td></td><td></td><td></td></tr>
<tr><td>7</td><td>117559479</td><td>G</td><td>A</td><td>0|1</td><td>G</td><td>A</td><td>rs213950</td><td>c.1408G>A</td><td>p.Val470Met</td><td>0.418961</td><td>|</td><td></td><td></td><td></td></tr>
<tr><td>7</td><td>117560427</td><td>G</td><td>C</td><td>0|1</td><td>G</td><td>C</td><td>rs1896887</td><td>c.1584+772G>C</td><td></td><td>0.417086</td><td>|</td><td>J7-8</td><td>F7-8_J_s1</td><td>TATGCATATGAACCCTTCACACTACCC</td></tr>
<tr><td>7</td><td>117560456</td><td>G</td><td>T</td><td>0|1</td><td>G</td><td>T</td><td>rs1896886</td><td>c.1584+801G>T</td><td></td><td>0.417474</td><td>|</td><td>|</td><td></td><td></td></tr>
<tr><td>7</td><td>117562430</td><td>C</td><td>G</td><td>0|1</td><td>C</td><td>G</td><td>rs2283057</td><td>c.1584+2775C>G</td><td></td><td>0.417344</td><td>PCR-7</td><td>|</td><td></td><td></td></tr>
<tr><td>7</td><td>117567806</td><td>C</td><td>A</td><td>0|1</td><td>C</td><td>A</td><td>rs718829</td><td>c.1584+8151C>A</td><td></td><td>0.420543</td><td>PCR-8</td><td>|</td><td></td><td></td></tr>
<tr><td>7</td><td>117571314</td><td>C</td><td>T</td><td>1|0</td><td>T</td><td>C</td><td>rs183421277</td><td>c.1584+11659C>T</td><td></td><td>0.011180</td><td>|</td><td>|</td><td></td><td></td></tr>
<tr><td>7</td><td>117571799</td><td>T</td><td>C</td><td>0|1</td><td>T</td><td>C</td><td>rs2082056</td><td>c.1584+12144T>C</td><td></td><td>0.422464</td><td>|</td><td>J7-8</td><td>F7-8_J_as1</td><td>ATACATGTGAACGTGTATCTGAGAGTG</td></tr>
<tr><td>7</td><td>117572669</td><td>T</td><td>C</td><td>0|1</td><td>T</td><td>C</td><td>rs213934</td><td>c.1584+13014T>C</td><td></td><td>0.422808</td><td>|</td><td>J8-9</td><td>F8-9_J_s2</td><td>CACACACTCTACTCTTCCCTTCCTCTG</td></tr>
<tr><td>7</td><td>117574035</td><td>T</td><td>C</td><td>0|1</td><td>T</td><td>C</td><td>rs3808185</td><td>c.1585-13704T>C</td><td></td><td>0.416117</td><td>|</td><td>|</td><td></td><td></td></tr>
<tr><td>7</td><td>117574071</td><td>T</td><td>C</td><td>0|1</td><td>T</td><td>C</td><td>rs3808184</td><td>c.1585-13668T>C</td><td></td><td>0.418605</td><td>PCR-8</td><td>|</td><td></td><td></td></tr>
<tr><td>7</td><td>117577667</td><td>C</td><td>T</td><td>0|1</td><td>C</td><td>T</td><td>rs2518873</td><td>c.1585-10072C>T</td><td></td><td>0.421960</td><td>PCR-9</td><td>|</td><td></td><td></td></tr>
<tr><td>7</td><td>117578105</td><td>T</td><td>A</td><td>0|1</td><td>T</td><td>A</td><td>rs2402228</td><td>c.1585-9634T>A</td><td></td><td>0.420848</td><td>|</td><td>|</td><td></td><td></td></tr>
<tr><td>7</td><td>117578521</td><td>G</td><td>A</td><td>0|1</td><td>G</td><td>A</td><td>rs213953</td><td>c.1585-9218G>A</td><td></td><td>0.422898</td><td>|</td><td>|</td><td></td><td></td></tr>
<tr><td>7</td><td>117579781</td><td>C</td><td>T</td><td>0|1</td><td>C</td><td>T</td><td>rs1469486</td><td>c.1585-7958C>T</td><td></td><td>0.420392</td><td>|</td><td>|</td><td></td><td></td></tr>
<tr><td>7</td><td>117580630</td><td>G</td><td>A</td><td>0|1</td><td>G</td><td>A</td><td>rs4148709</td><td>c.1585-7109G>A</td><td></td><td>0.414530</td><td>|</td><td>J8-9</td><td>F8-9_J_as1</td><td>GAAACCTGCCTTCAAGCACTTCTGTGC</td></tr>
<tr><td>7</td><td>117580807</td><td>A</td><td>G</td><td>0|1</td><td>A</td><td>G</td><td>rs213955</td><td>c.1585-6932A>G</td><td></td><td>0.421379</td><td>|</td><td></td><td></td><td></td></tr>
<tr><td>7</td><td>117580952</td><td>G</td><td>A</td><td>0|1</td><td>G</td><td>A</td><td>rs213956</td><td>c.1585-6787G>A</td><td></td><td>0.421553</td><td>|</td><td></td><td></td><td></td></tr>
<tr><td>7</td><td>117582184</td><td>A</td><td>G</td><td>0|1</td><td>A</td><td>G</td><td>rs6466614</td><td>c.1585-5555A>G</td><td></td><td>0.414093</td><td>|</td><td></td><td></td><td></td></tr>
<tr><td>7</td><td>117583536</td><td>A</td><td>G</td><td>0|1</td><td>A</td><td>G</td><td>rs213957</td><td>c.1585-4203A>G</td><td></td><td>0.422227</td><td>|</td><td>J9-10</td><td>F9-10_J_s1</td><td>GGAGGAGAGTACTGTCTCTTATCAGCC</td></tr>
<tr><td>7</td><td>117583710</td><td>G</td><td>A</td><td>0|1</td><td>G</td><td>A</td><td>rs213958</td><td>c.1585-4029G>A</td><td></td><td>0.421997</td><td>|</td><td>|</td><td></td><td></td></tr>
<tr><td>7</td><td>117584386</td><td>G</td><td>A</td><td>1/1</td><td>A</td><td>A</td><td>rs213960</td><td>c.1585-3353G>A</td><td></td><td>1.000000</td><td>|</td><td>|</td><td></td><td></td></tr>
<tr><td>7</td><td>117584670</td><td>C</td><td>A</td><td>0|1</td><td>C</td><td>A</td><td>rs213961</td><td>c.1585-3069C>A</td><td></td><td>0.422777</td><td>|</td><td>|</td><td></td><td></td></tr>
<tr><td>7</td><td>117585105</td><td>C</td><td>T</td><td>0|1</td><td>C</td><td>T</td><td>rs6949974</td><td>c.1585-2634C>T</td><td></td><td>0.418641</td><td>PCR-9</td><td>|</td><td></td><td></td></tr>
<tr><td>7</td><td>117589113</td><td>G</td><td>A</td><td>0|1</td><td>G</td><td>A</td><td>rs213963</td><td>c.1680-1240G>A</td><td></td><td>0.422136</td><td>PCR-10</td><td>|</td><td></td><td></td></tr>
<tr><td>7</td><td>117589282</td><td>A</td><td>G</td><td>0|1</td><td>A</td><td>G</td><td>rs213964</td><td>c.1680-1071A>G</td><td></td><td>0.421929</td><td>|</td><td>|</td><td></td><td></td></tr>
<tr><td>7</td><td>117589483</td><td>T</td><td>A</td><td>0|1</td><td>T</td><td>A</td><td>rs213965</td><td>c.1680-870T>A</td><td></td><td>0.422184</td><td>|</td><td>|</td><td></td><td></td></tr>
<tr><td>7</td><td>117589765</td><td>A</td><td>G</td><td>0|1</td><td>A</td><td>G</td><td>rs213966</td><td>c.1680-588A>G</td><td></td><td>0.421512</td><td>|</td><td>|</td><td></td><td></td></tr>
<tr><td>7</td><td>117589783</td><td>G</td><td>T</td><td>0|1</td><td>G</td><td>T</td><td>rs213967</td><td>c.1680-570G>T</td><td></td><td>0.420675</td><td>|</td><td>|</td><td></td><td></td></tr>
<tr><td>7</td><td>117589939</td><td>C</td><td>T</td><td>0|1</td><td>C</td><td>T</td><td>rs213968</td><td>c.1680-414C>T</td><td></td><td>0.423032</td><td>|</td><td>|</td><td></td><td></td></tr>
<tr><td>7</td><td>117590591</td><td>T</td><td>A</td><td>0|1</td><td>T</td><td>A</td><td>rs4148711</td><td>c.1766+152T>A</td><td></td><td>0.418447</td><td>|</td><td>|</td><td></td><td></td></tr>
<tr><td>7</td><td>117590726</td><td>T</td><td>C</td><td>0|1</td><td>T</td><td>C</td><td>rs3808183</td><td>c.1766+287T>C</td><td></td><td>0.415153</td><td>|</td><td>J9-10</td><td>F9-10_J_as1</td><td>AGTCCTGAGCTCTGTGTGACTGAGGAG</td></tr>
<tr><td>7</td><td>117591092</td><td>C</td><td>T</td><td>1/1</td><td>T</td><td>T</td><td>rs213969</td><td>c.1766+653C>T</td><td></td><td>1.000000</td><td>|</td><td></td><td></td><td></td></tr>
<tr><td>7</td><td>117593640</td><td>G</td><td>C</td><td>0|1</td><td>G</td><td>C</td><td>rs213970</td><td>c.2490+983G>C</td><td></td><td>0.422631</td><td>|</td><td></td><td></td><td></td></tr>
<tr><td>7</td><td>117594122</td><td>G</td><td>A</td><td>0|1</td><td>G</td><td>A</td><td>rs213971</td><td>c.2491-808G>A</td><td></td><td>0.423660</td><td>|</td><td></td><td></td><td></td></tr>
<tr><td>7</td><td>117594483</td><td>A</td><td>G</td><td>0|1</td><td>A</td><td>G</td><td>rs117243</td><td>c.2491-447A>G</td><td></td><td>0.422317</td><td>|</td><td></td><td></td><td></td></tr>
<tr><td>7</td><td>117595001</td><td>T</td><td>G</td><td>0|1</td><td>T</td><td>G</td><td>rs1042077</td><td>c.2562T>G</td><td>p.Thr854Thr</td><td>0.417215</td><td>|</td><td>J10-11</td><td>F10-11_J_s2</td><td>GAGAGCATACCAGCAGTGACTACATGG</td></tr>
<tr><td>7</td><td>117595164</td><td>T</td><td>A</td><td>0|1</td><td>T</td><td>A</td><td>rs4148713</td><td>c.2619+106T>A</td><td></td><td>0.411948</td><td>|</td><td>|</td><td></td><td></td></tr>
<tr><td>7</td><td>117595496</td><td>A</td><td>G</td><td>0|1</td><td>A</td><td>G</td><td>rs6979652</td><td>c.2619+438A>G</td><td></td><td>0.418125</td><td>|</td><td>|</td><td></td><td></td></tr>
<tr><td>7</td><td>117595832</td><td>A</td><td>G</td><td>0|1</td><td>A</td><td>G</td><td>rs213973</td><td>c.2619+774A>G</td><td></td><td>0.420085</td><td>|</td><td>|</td><td></td><td></td></tr>
<tr><td>7</td><td>117596212</td><td>T</td><td>C</td><td>0|1</td><td>T</td><td>C</td><td>rs213974</td><td>c.2619+1154T>C</td><td></td><td>0.420610</td><td>|</td><td>|</td><td></td><td></td></tr>
<tr><td>7</td><td>117597224</td><td>C</td><td>T</td><td>0|1</td><td>C</td><td>T</td><td>rs76258137</td><td>c.2619+2166C>T</td><td></td><td>0.416474</td><td>|</td><td>|</td><td></td><td></td></tr>
<tr><td>7</td><td>117597274</td><td>C</td><td>T</td><td>0|1</td><td>C</td><td>T</td><td>rs2078753</td><td>c.2619+2216C>T</td><td></td><td>0.413346</td><td>PCR-10</td><td>|</td><td></td><td></td></tr>
<tr><td>7</td><td>117606701</td><td>A</td><td>C</td><td>0|1</td><td>A</td><td>C</td><td>rs397508462</td><td>c.2936A>C</td><td>p.Asp979Ala</td><td>0.000386</td><td>PCR-11</td><td>J10-11</td><td>F10-11_J_as1</td><td>GGTTTGGGCCAGGTAAGCAGTTCTGAC</td></tr>
</table>

Supplementary Table S5: Characteristics of Patient BM1182 with pathogenic *CFTR* variants detected by sequencing of the entire gene

Item	Characteristics
**Demographic information**	
Place of recruitment	Department of Ear, Nose, and Throat
Gender	Female
Age (y.o.)	28.5
Body mass index	20.8
Hospitalized	No
Ethnicity	Kinh
Marital status	Married, 2 years
No. of children	0
**Medical/family history**	
Diagnosis of CRS	Yes, 10 years ago
Concomittent disease	GERD, stenosis of the uterus cervix
Disease history	Pneumonia at 10 y.o.
History of contact	No
Duration of contact	0
Smoking	No
History of allergy	No
Allergic symtoms	No
Long term low dose macrolide treatment	No
Disease history of family members	Husband with bronchiectasis and chronic bronchitis
**Physical findings**	
Productive cough	Yes
Age of onset (persistent productive cough) (y.o.)	9.5
Duration of productive cough	19 years
Hemoptysis	Yes
Dyspnoea	Yes, when walking up an incline
Duration of dyspnoea	5 years
Duration of nasal obstruction	20 years
Duration of nasal discharge	20 years
Duration of facial pain	10 years
Duration of reduction or loss of smell	10 years
Body temperature (^{o}C)	37.2
Pulse rate (per min)	90
Blood pressure (mmHg)	90/60
Clubbed finger	No
Cyanosis	No
Barrel chest	No
Funnel chest	No
Ausculation	crackles, wheeze
**Sputum culture**	Negative
**Blood test**	
CRP (mg/dl)	0.39
IgE (IU/ml)	13.4
Blood eosinophil count (cells/µl)	100
Rheumatic factor (U/ml)	6.1

**Pulmonary function test**	
VC (liter)	3.12
FEV1 (liter)	2.68
FVC (liter)	3.16
FEV1/FVC ratio	0.85
**Otolaryngoscopic findings**	
Mucopurulent from middle meatus	Yes
Edema or mucosal obstruction in middle meatus	Yes
Presence of nasal polyps	No
**Chest CT**	
CT main findings	Presence of bronchiectasis (cylindrical, mild, 4/6 zones: right upper, right middle, left middle, left lower), bronchitis and bronchiolar lesions (centrilobular nodular, tree-in-bud sign, and bronchiolectasis; 3/6 zones: right upper, right middle, left lower)
**Situs inversus**	No

CRS: chronic rhinosinusitis, GERD: gastroesophageal reflux disease, CT: computed tomography, VC: vital capacity, FEV1: forced expiratory volume in one second, FVC: forced vital capacity.

Supplementary Table S6: Benign, likely-benign, or variants of uncertain significance identified by sequencing of the entire *CFTR* gene among cases with extensive BL/BE and early-onset, persistent productive cough (n = 7)

**Patient ID**	**Gender**	**Age**	**Age of onset (persistent productive cough)**	**Duration of cough (years)**	**Variant description**	**Zygosity**	**Classification in ClinVar***	TGmTn allele 1	TmTGn allele 2
BM1182	F	28.5	9.5	19	chr7:117559479; NM_000492.4:c.1408G>A (p.Val470Met)	Heterozygous	Benign/Likely benign	TG12T7	TG12T7
BM2094	F	17.9	14.9	3	chr7:117559479; NM_000492.4:c.1408G>A (p.Val470Met)	Heterozygous	Benign/Likely benign	TG11T7	TG12T7
BM2130	F	28.9	19.9	9	chr7: 117587820; NM_000492.4:c.1666A>G (p.Ile556Val)	Heterozygous	Conflicting classifications of pathogenicity<br>Pathogenic(1); Uncertain significance(1);<br>Benign(4); Likely benign(5)	TG11T7	TG12T5
BM1050	M	25.5	15.5	10	chr7:117559479; NM_000492.4:c.1408G>A (p.Val470Met)	Heterozygous	Benign/Likely benign	TG12T7	TG12T7
BM1194	F	23.0	20.0	3	chr7:117559479; NM_000492.4:c.1408G>A (p.Val470Met)	Heterozygous	Benign/Likely benign	TG11T7	TG12T7
BM2026	F	18.4	13.4	5	chr7:117559479; NM_000492.4:c.1408G>A (p.Val470Met)	Heterozygous	Benign/Likely benign	TG11T7	TG12T7
					chr7: 117664780; NM_000492.4:c.4056G>C (p.Gln1352His)	Heterozygous	Conflicting classifications of pathogenicity:<br>Pathogenic(2); Uncertain significance(10);<br>Benign(1); Likely benign(2)		
BM2046	F	26.6	16.5	10	chr7: 117587820; NM_000492.4:c.1666A>G (p.Ile556Val)	Heterozygous	Conflicting classifications of pathogenicity<br>Pathogenic(1); Uncertain significance(1);<br>Benign(4); Likely benign(5)	TG11T7	TG12T5

M: male, F: female, BL: bronchiolar lesions, BE: bronchiectasis
*https://www.ncbi.nlm.nih.gov/clinvar/

Supplementary Table S7: Characteristics of patients suspected of PCD with or without identified causative genes using targeted resequencing of 42 PCD-causative genes

Characteristics	BM1139*	BM1179*	BM2118	BM2094	BM2130	BM2045**
**Laterality defect (situs inversus)**	Yes (liver is in the normal position)	Yes	Yes	No	No	No
**PCD-causing genes**	NA	NA	*DNAAF11 (LRRC6)*	*RSPH1*	*RSPH1*	*CCDC39*
**Demographic information**						
Place of recruitment	Department of Ear, Nose, and Throat	Department of Ear, Nose, and Throat	Respiratory Center	Respiratory Center	Respiratory Center	Respiratory Center
Gender	Female	Female	Male	Female	Female	Male
Age (y.o.)	20.8	14.6	28.0	17.90	28.99	36.68
Body mass index	17.3	19.6	23.1	18.90	23.30	23.6
Hospitalized	No	No	No	No	No	Yes
Ethnicity	Kinh	Kinh	Kinh	Kinh	Kinh	Kinh
Marital status	Single	Single	Married	Single	Married	Married
No. of children	0	0	3	0	0	2
**Medical/family history**						
Diagnosis of CRS	Yes, 10 years ago	Yes, 5 years ago	Yes, this time	Yes, 2 years ago	Yes, 3 years ago	Yes, 15 years ago
Concomittent disease	Asthma	No	GERD	No	No	Asthma
Disease history	Pneumonia at 6 months old, measles at 4 y.o.	Pneumonia at 12 y.o., measles at 5 y.o.	No	No	Pertusis at 6 y.o., meales at 6 y.o.	Pneumonia
History of contact	No	No	Anti-cancer chemical	No	No	Yes, with gasoline
Duration of contact	0	0	2 years	0	0	NA
Smoking	No	No	No	No	No	No
History of allergy	Food, weather	Not clear	Medicine	No	No	No
Allergic symtoms	Sneezing, dyspnoea, cough	Rash, urticartia	Rash	No	No	No
Long term low dose macrolide treatment	No	No	No	No	No	No
Disease history of family members and age of onset	Sibling with CRS: 10 y.o.	Sibling with CRS: 9 y.o., bronchiectasis: 19 y.o.	Parent with CRS: 50 y.o.	No	Grandfather/granmother with COPD: 40 y.o.	No
**Physical findings**						
Productive cough	Yes	Yes	Yes	Yes	Yes	Yes
Age of onset (persistent productive cough) (y.o.)	4 months old	9.6	8.0	15.0	20.0	26
Duration of productive cough	20 years	5 years	20 years	3 years	9 years	10 years
Hemoptysis	Yes	No	No	No	Yes	No
Dyspnoea	Yes, when walking up an incline	Yes, when walking up an incline	No	Yes, when walking up an incline	No	Yes, still dyspnoea even after stop walking
Duration of dyspnoea	3 years	3 years	NA	3 years	0.00	3 years
Duration of nasal obstruction	12 years	7 years	20 years	3 years	5 years	20 years
Duration of nasal discharge	12 years	7 years	20 years	3 years	5 years	20 years
Duration of facial pain	3 years	2 years	10 years	3 years	3 years	NA
Duration of reduction or loss of smell	3 years	1 year	10 years	0	3 years	20 years
Body temperature (^{o}C)	36.9	36.8	36.7	37.0	36.6	38
Pulse rate (per min)	84	90	77	80	80	100
Blood pressure (mmHg)	110/70	100/60	90/60	100/70	100/60	110/70
Clubbed finger	No	No	No	No	No	Yes
Cyanosis	No	No	No	No	No	Yes
Barrel chest	No	No	No	No	No	Yes
Funnel chest	No	No	No	No	No	No
Ausculation	Crackles, wheeze	Normal	Crackles, wheeze	crackles, wheeze	crackles, wheeze	crackles, wheeze
**Sputum culture**	Negative	Negative	Negative	Positive (*Streptococcus pneumoniae*)	Negative	Negative
**Blood test**						
CRP (mg/dl)	0.6	0.02	0.4	0.7	0.3	2.8

IgE (IU/ml)	1029	388.2	15.8	2.2	155.5	73
Blood eosinophil count (cells/μl)	40	100	240	240	290	0
Rheumatic factor (U/ml)	12.5	11.2	15.2	17.0	16.5	9.9
**Pulmonary function test**						
VC (liter)	2.21	2.45	4.37	2.62	2.41	NA
FEV1 (liter)	0.9	1.54	2.84	2.01	1.76	0.77
FVC (liter)	2.01	2.73	4.34	2.63	2.48	1.76
FEV1/FVC ratio	0.45	0.56	0.65	0.76	0.71	0.44
**Otolaryngoscopic findings**						
Mucopurulent from middle meatus	Yes	Yes	Yes	Yes	Yes	Yes
Edema or mucosal obstruction in middle meatus	Yes	Yes	Yes	Yes	Yes	Yes
Presence of nasal polyps	No	No	No	No	Yes	No
**Chest CT**						
CT main findings (type, severity, location in the lung zones)	Presence of bronchiectasis (cylindrical, moderate, 3/6 zones: right middle, left middle, left lower), bronchitis and bronchiolar lesions (1/6 zone: right middle)	Presence of bronchiectasis (cylindrical, mild, 2/6 zones: right lower, left middle), bronchitis and bronchiolar lesions (centrilobular nodular, tree-in-bud sign, and bronchiolectasis, 2/6 zones: right lower, left middle)	Presence of bronchiectasis (varicose, moderate, 1/6 zone: left middle), bronchitis and bronchiolar lesions (centrilobular nodular, mild, 2/6 zones: right upper, left lower)	Presence of bronchiectasis (cylindrical, mild, 2/6 zones: right middle, left middle), bronchitis and bronchiolar lesions (centrilobular nodular, tree-in-bud sign, and bronchiolectasis, 4/6: right middle, right lower, left middle, left lower)	Presence of bronchiectasis (varicose, severe, 5/6 zones except left upper), bronchitis, bronchiolar lesions (centrilobular nodular, tree-in-bud sign, and bronchiolectasis, 4/6: right middle, right lower, left middle, left lower)	Presence of bronchiectasis (cylindrical, moderate, 5/6 zones except left upper), bronchitis, bronchiolar lesions (centrilobular nodular, tree-in-bud sign, and bronchiolectasis; 6/6 zones)

* Sisters with each other
PCD: Primary ciliary dyskinesia, CRS: chronic rhinosinusitis, VC: vital capacity, FEV1: forced expiratory volume in one second, FVC: forced vital capacity, CT: computed tomography, GERD: gastroesophageal reflux disease, NA: not available
**Although this patient was not initially a candidate for PCD screening, genetic testing prompted by low IgG levels incidentally identified a PCD-causing variant.

Supplementary Table S8: *CFTR* variants possibly associated with extensive BL/BE

**Gene and variants**	**Diffuse BL/BE* (1)**	**Other/no CT abnormalities* (2)**	**Healthy control** (3)**	**Uncorrected P, compared between (1) and (2)**	**Uncorrected P compared between (1) and (3)**
**Poly-T variants**	2n=54	2n=336	2n=990	> 0.999	0.805
c.1210-12T[5] (T5)	1 (0.019)	6 (0.018)	37 (0.037)		
c.1210-12T[7] (T7)	53 (0.981)	329 (0.979)	946 (0.946)		
c.1210-12T[9] (T9)	0 (0.000)	1 (0.003)	6 (0.006)		
c.1210-12T[6] (T6)	0 (0.000)	0 (0.000)	1 (0.001)		
**TG-Repeat Polymorphisms**	2n=54	2n=326	2n=990	0.960	0.910
TG10	0 (0.000)	1 (0.003)	6 (0.006)		
TG11	24 (0.444)	150 (0.460)	406 (0.410)		
TG12	29 (0.537)	170 (0.522)	557 (0.563)		
TG13	1 (0.019)	5 (0.015)	21 (0.021)		
**Met470Val**					
Genotype	n=27	n=168	n=495	0.908	0.801
AA (Met/Met)	6 (22.2%)	33 (19.6%)	106 (21.4%)		
AG (Met/Val)	12 (44.5%)	82 (48.8%)	250 (50.5%)		
GG (Val/Val)	9 (33.3%)	53 (31.6%)	139 (28.1%0		
Allele	2n=54	2n=336	2n=990	0.957	0.750
A (Met)	24 (0.444)	148 (0.440)	462 (0.467)		
G (Val)	30 (0.556)	188 (0.560)	528 (0.533)		

*Current study population, after excluding one cystic fibrosis and three primary ciliary dyskinesia genetically confirmed cases and one in which DNA was unavailable

**495 Vietnamese healthy controls (Nam MH, Am J Med Genet A. 2005)

BL: bronchiolar lesions, BE: bronchiectasis, CT: computed tomography

Supplementary Table S9: Frequencies of HLA alleles† in extensive BL/BE compared with controls

**HLA locus**	**Diffuse BL/BE¶ (2n = 54) (1)**	**Other/no CT abnormalities¶ (2n = 336) (2)**	**Healthy control§ (2n = 340) (3)**	**Uncorrected P value** [compared between (1) and (2)]	**Uncorrected P value** [compared between (1) and (3)]
HLA-A					
A*02:03	0.093	0.077	0.079	NS	NS
A*02:06	0.056	0.027	0.047	NS	NS
A*02:07	0.093	0.110	0.085	NS	NS
A*11:01	0.241	0.286	0.229	NS	NS
A*11:02	0.019	0.063	0.035	NS	NS
A*24:02	0.278	0.107	0.138	**0.001**	0.009
A*24:07	0.019	0.030	0.044	NS	NS
A*26:01	0.000	0.045	0.021	NS	NS
A*29:01	0.019	0.077	0.062	NS	NS
A*33:03	0.071	0.066	0.115	NS	NS
HLA-B					
B*07:05	0.093	0.083	0.074	NS	NS
B*13:01	0.037	0.048	0.038	NS	NS
B*15:02	0.13	0.131	0.135	NS	NS
B*15:25	0.019	0.048	0.059	NS	NS
B*38:02	0.019	0.048	0.056	NS	NS
B*40:01	0.056	0.083	0.062	NS	NS
B*44:03	0.037	0.009	0.038	NS	NS
B*46:01	0.167	0.140	0.115	NS	NS
B*54:01	0.093	0.027	0.024	0.016	0.008
B*55:02	0.056	0.045	0.026	NS	NS
B*58:01	0.056	0.045	0.065	NS	NS
HLA-DRB1					
DRB1*04:03	0.019	0.045	0.021	NS	NS
DRB1*04:05	0.148	0.066	0.038	0.034	**0.001**
DRB1*07:01	0.056	0.051	0.076	NS	NS
DRB1*08:03	0.093	0.042	0.041	NS	NS

DRB1*09:01	0.148	0.128	0.097	NS	NS
DRB1*10:01	0.056	0.060	0.056	NS	NS
DRB1*12:02	0.241	0.271	0.353	NS	NS
DRB1*13:02	0.037	0.012	0.018	NS	NS
DRB1*13:03	0.074	0.036	0.018	NS	0.035
DRB1*15:01	0.019	0.048	0.038	NS	NS
DRB1*15:02	0.037	0.083	0.062	NS	NS

† Ten HLA alleles with the highest frequencies in each locus, and those reported to be associated with DPB in Japan (Keicho, Am J Respir Crit Care Med. 1998) are shown.

¶ Current study population, after excluding one case in which the DNA sample was unavailable; one cystic fibrosis and three primary ciliary dyskinesia genetically confirmed cases.

§ 170 Vietnamese healthy control (Hoa BK, Tissue Antigen. 2008)

BL: bronchiolar lesions, BE: bronchiectasis, CT: computed tomography, **bold** shows significant P value after Bonferroni correction, NS: not significant

Supplementary Table S10: Genes and single nucleotide variants possibly associated with extensive BL/BE*

Gene and variants	Diffuse BL/BE** (1)	Other/no CT abnormalities** (2)	Healthy control*** (3)	Uncorrected P value, compared between (1) and (2)	Uncorrected P value, compared between (1) and (3)
***MUC22* (*PBMUCL1*) intron 2 (rs117121291)**					
Genotype	n = 27	n = 168	n = 168	**0.008**	**0.009**
CC	22 (81.5%)	160 (95.2%)	162 (96.4%)		
CT	5 (18.5%)	8 (4.8%)	6 (3.6%)		
TT	0 (0.0%)	0 (0.0%)	0 (0.0%)		
Allele	2n = 54	2n = 336	2n = 336	**0.009**	**0.002**
C	49 (0.907)	328 (0.976)	330 (0.982)		
T	5 (0.093)	8 (0.024)	6 (0.018)		
***MUC22* (*PBMUCL1*) exon 5 (rs4248153)**					
Genotype	n = 27	n = 168	n = 506	0.755	0.235
AA	16 (59.3%)	87 (51.8%)	218 (43.1%)		
AG	10 (37.0%)	69 (41.1%)	230 (45.5%)		
GG	1 (3.7%)	12 (7.1%)	58 (11.5%)		
Allele	2n = 54	2n = 336	2n = 1012	0.401	0.070
A	42 (0.778)	243 (0.723)	666 (0.658)		
G	12 (0.222)	93 (0.277)	346 (0.342)		
***MUC5B* _promoter -717 (rs17235353)**					
Genotype	n = 27	n = 168	n = 506	0.852	0.695
II	16 (59.3%)	91 (54.2%)	255 (50.4%)		
DI	9 (33.3%)	65 (38.7%)	207 (40.9%)		
DD	2 (7.4%)	12 (7.1%)	44 (8.7%)		
Allele frequency	2n = 54	2n = 336	2n = 1012	0.708	0.423
I	41 (0.759)	247 (0.735)	717 (0.708)		
D	13 (0.241)	89 (0.265)	295 (0.292)		
***DEFB1* promoter region (rs1799946, rs1800972 and rs11362, located at positions -52, -44, -20 of the transcription start site)**					
Allele frequency	2n = 54	2n = 314	2n = 1012	0.442	0.619
GCA	29 (0.537)	147 (0.468)	477 (0.471)		
ACG	20 (0.370)	145 (0.462)	449 (0.444)		
GGG	5 (0.093)	22 (0.070)	80 (0.079)		
GCG	0 (0.000)	0 (0.000)	6 (0.006)		

*After excluding one cystic fibrosis and three primary ciliary dyskinesia genetically confirmed cases.
**Current study population, after excluding one cystic fibrosis and three primary ciliary dyskinesia genetically confirmed cases, and one in which DNA was unavailable.
***168 (Hoa BK, Tissue Antigen. 2008) and 506 Vietnamese healthy volunteers (Hijikata M. Hum Genet. 2012) were used as controls.
BL: bronchiolar lesions, BE: bronchiectasis, CT: computed tomography, I: insertion, D: deletion, **bold** shows significant P values

Supplementary Fig. S1
The pathogenic *CFTR* variants in Patient BM1182
The whole-*CFTR*-gene sequencing was performed using a next-generation sequencer, and the reads were mapped to the human reference genome GRCh38 to identify the variants (left panel). The variants were confirmed by PCR-direct Sanger sequencing (right panel). The results from a control sample are also shown. Arrows indicate the positions of the variants. **(a)** A heterozygous pathogenic variant NM_000492.4:c.1202G>A (p.Trp401Ter); chr7:117,542,101 G>A; rs397508174. **(b)** A heterozygous pathogenic variant NM_000492.4:c.2936A>C (p.Asp979Ala); chr7:117,606,701 A>C; rs397508462. PCR: polymerase chain reaction.

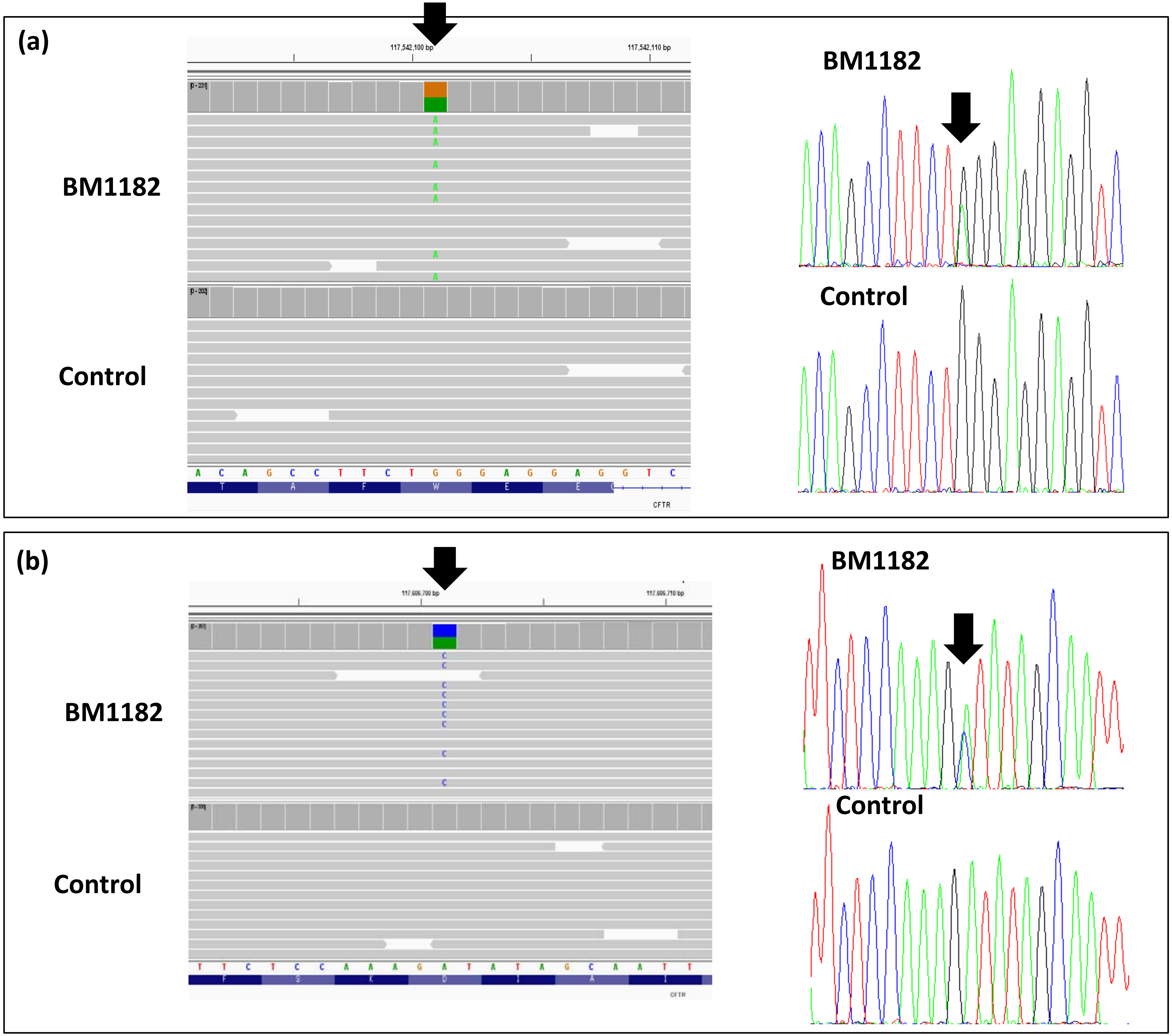

Supplementary Fig. S2
Overlapping long-range PCRs in the *CFTR* region and phased long reads in Patient BM1182
The genomic region spanning *CFTR*:c.1202G>A (p.Trp401Ter) and c.2936A>C (p.Asp979Ala) was amplified using 11 overlapping long-range PCRs (upper panel), sequenced with a long-read sequencer, and reads were aligned to the human reference genome. Haplotype tags were assigned to each read, and single nucleotide variants with the two haplotypes (haplotype 1 in light blue and haplotype 2 in pink) were visualized in IGV (lower panel). PCR: polymerase chain reaction, IGV: Integrative Genomics Viewer.

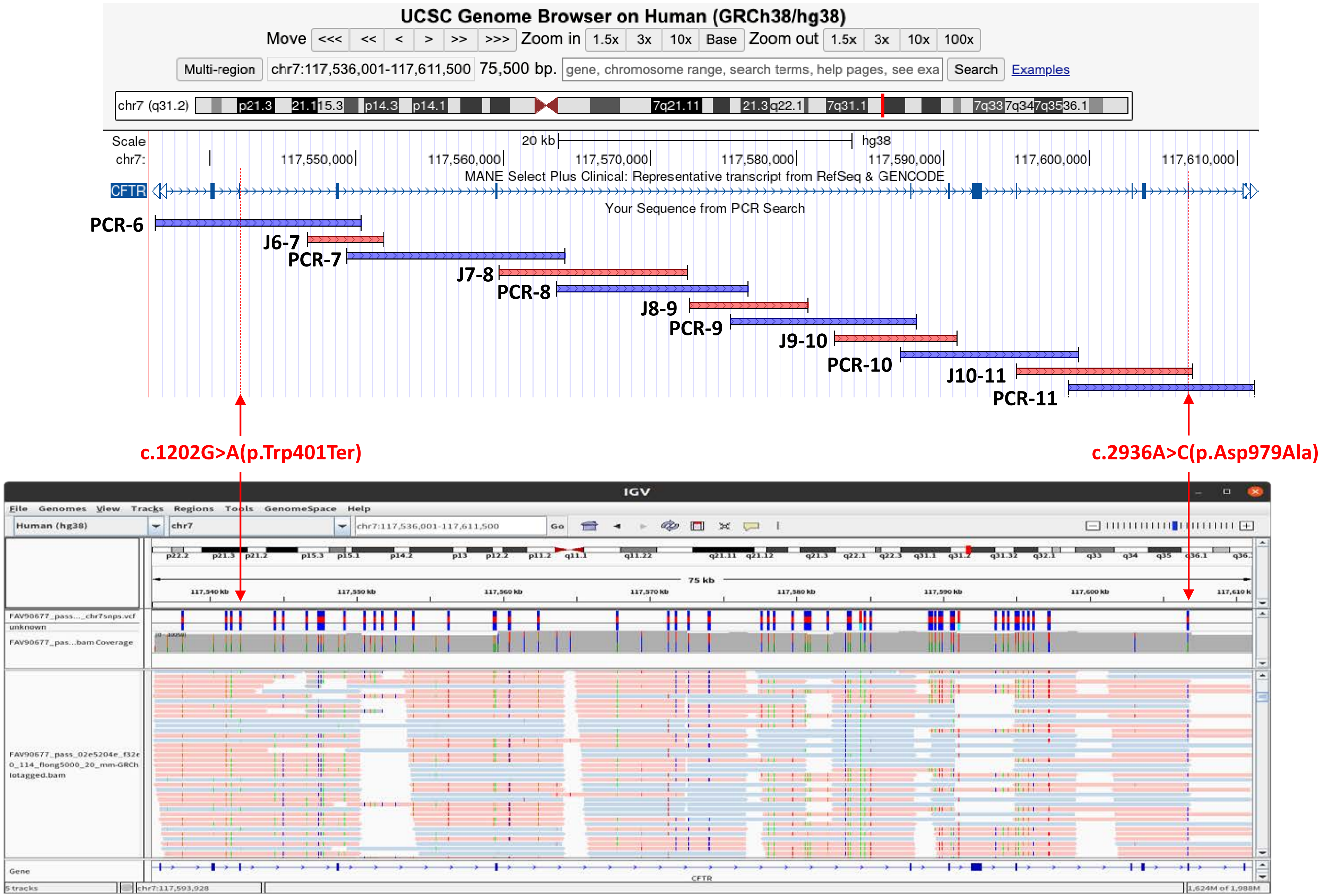

Haplotype 1
Haplotype 2

Supplementary Fig. S3

PCD-causing gene variants identified by next-generation sequencing (left panel) and PCR-direct Sanger sequencing (right panel)

(a) A homozygous/hemizygous pathogenic variant to disrupt the start codon of *DNAAF11* from ATG to GTG was identified in Patient BM2118. NM_012472.6:c.1A>G (NP_036604.2:p.Met1?); chr8: 132,675,493 T>C. (b) A homozygous/hemizygous pathogenic variant to disrupt splice donor sequence of intron 4 from GT to AT in the *RSPH1* gene of Patient BM2094: NM_080860.4:c.365+1G>A; chr21:42,486,370 C>T. (c) A homozygous/hemizygous pathogenic variant, a four-nucleotide deletion causing a frameshift and premature stop codon in *RSPH1* of Patient BM2130: NM_080860.4:c.407_410del (NP_543136.1:p.Lys136MetfsTer6).

PCD: primary ciliary dyskinesia, PCR: polymerase chain reaction.

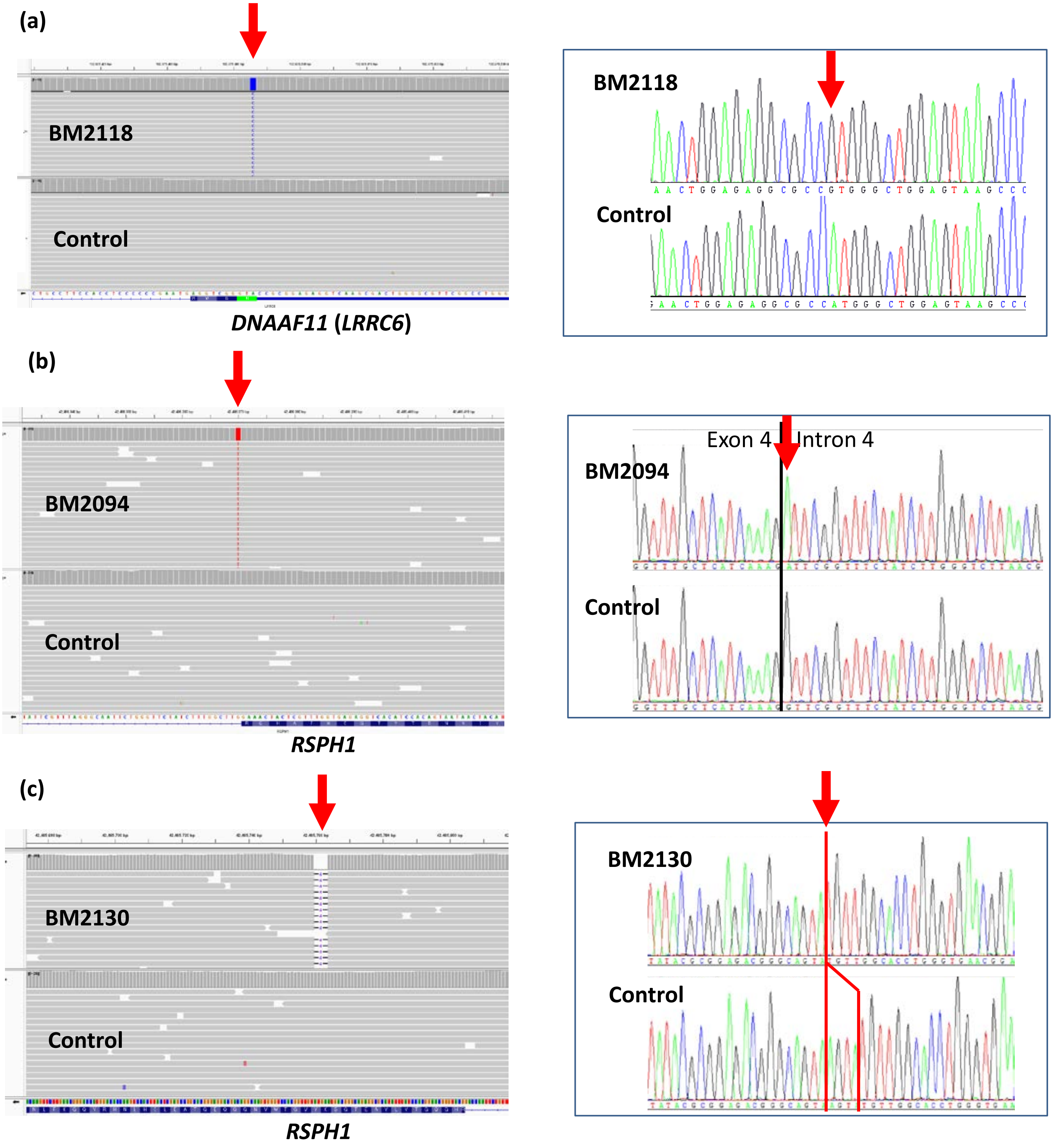

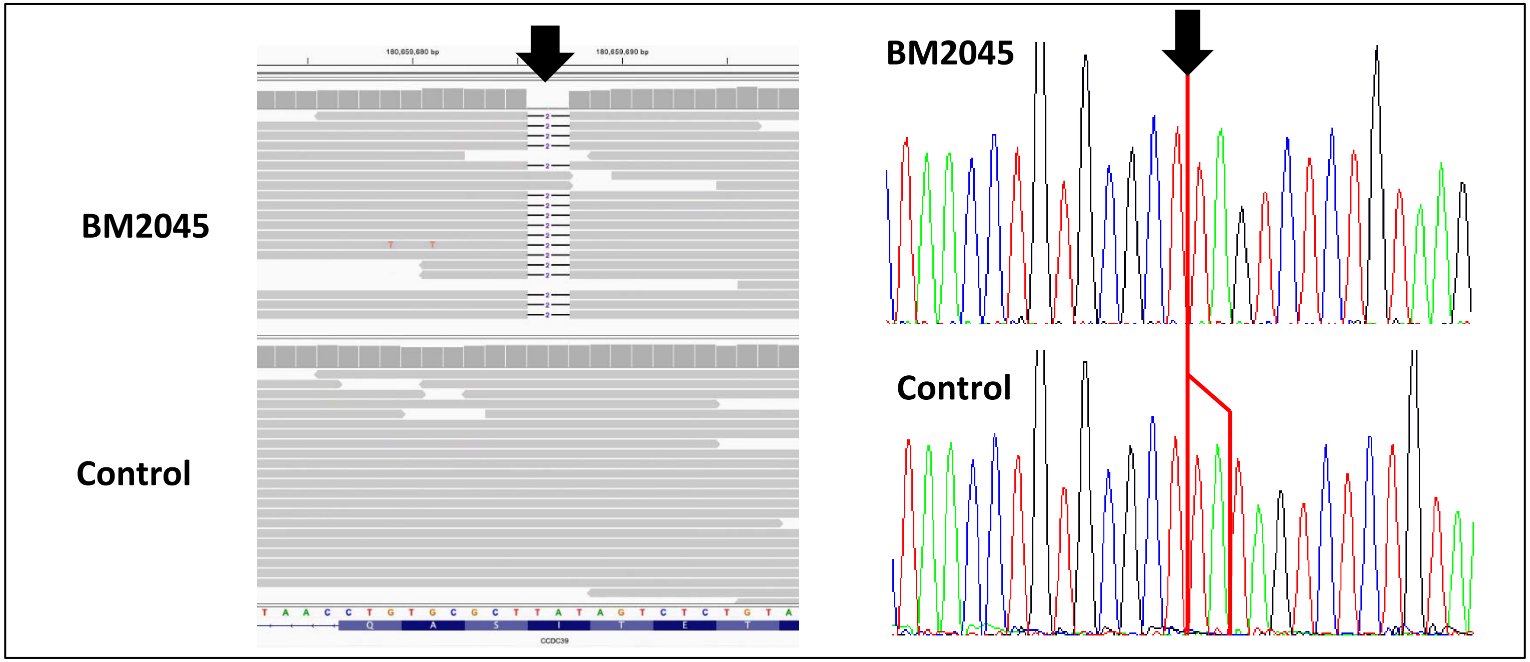

Supplementary Fig. S4
PCD-causing *CCDC39* variant in Patient BM2045

PCD-causing gene variants identified by next-generation sequencing (left panel) and PCR-direct Sanger sequencing (right panel) in Patient BM2045, compared with a control sample. Arrows indicate the positions of the homozygous/hemizygous pathogenic variant, a two-nucleotide deletion that causes a frameshift and premature stop codon in *CCDC39*: NM_181426.2:c.599_600del (NP_852091.1:p.Ile200LysfsTer8).
PCD: primary ciliary dyskinesia, PCR: polymerase chain reaction.
